# The impact of calorie labelling and proportional pricing on out of home food orders: a randomised controlled trial study using a virtual food and drink delivery app

**DOI:** 10.1186/s12966-023-01513-2

**Published:** 2023-09-19

**Authors:** Amy Finlay, Emma Boyland, Andrew Jones, Rozemarijn Witkam, Eric Robinson

**Affiliations:** 1 Department of Psychology, University of Liverpool, Liverpool, L69 7ZA UK; 2 Department of Psychology, Liverpool John Moores University, Liverpool, L3 3AF UK

**Keywords:** Calorie labelling, Proportional pricing, Food ordering, Out-of-home food, Food delivery apps

## Abstract

**Background:**

Mandatory calorie labelling in the out-of-home food sector was introduced in England in 2022, and menu pricing strategies that ensure cost is equivalent to portion size (proportional pricing) have been proposed as a policy to reduce obesity. Food delivery app-based platforms now contribute significantly to diet, and evidence suggests that those at a socioeconomic disadvantage may have greater exposure to unhealthy options on these platforms. However, public health policies to improve nutritional quality of food ordered from food delivery apps has received limited examination.

**Objective:**

This experimental study assessed the impact of calorie labelling and proportional pricing on item and meal size selection, calories ordered, and money spent when selecting food and drinks from three outlet types on a virtual delivery app.

**Methods:**

UK adult participants (N = 1126, 49% female), stratified by gender and education level completed an online study where they ordered items from three branded food and beverage outlets (coffee shop, sandwich outlet, fast food outlet) using a virtual delivery app. Participants were presented food and beverage options with vs. without calorie labels and with value (larger portions are proportionally cheaper) vs. proportional pricing.

**Results:**

Calorie labelling did not influence portion size selection for any outlets, but significantly reduced calories ordered from the coffee shop (-18.95kcals, 95% CI -33.07 to -4.84) and fast food outlet (-54.19kcals, 95% CI -86.04 to -22.33). Proportional pricing reduced the likelihood of choosing a larger beverage from the coffee shop (OR = 0.58, 95% CI 0.45 to 0.75), but was associated with increased calories ordered from the fast food outlet (51.25kcals, 95% CI 19.59 to 82.90). No consistent interactions were observed with participant characteristics, suggesting that effects of calorie labelling and pricing on outcomes were similar across sociodemographic groups.

**Conclusions:**

Calorie labelling on food delivery platforms may effectively reduce calories ordered. Proportional pricing may be useful in prompting consumers to select smaller portion sizes, although further research in real-world settings will now be valuable.

**Supplementary Information:**

The online version contains supplementary material available at 10.1186/s12966-023-01513-2.

## Introduction

Globally, there has been an increase in the presence, and use of food delivery platforms [1]. Uber Eats has shown an increase in the number of users from 5 million in 2016 to 66 million in 2020 [2]. Food delivery platforms are used most frequently in the UK by young adults [3, 4], and there is evidence that individuals with a lower educational level in the UK (high school completion or lower) use these platforms more than individuals with a higher (university degree or higher) or medium (some post-high school qualifications) level of education [4].

Food prepared outside the home, whether from fast food, takeaway, or full service restaurants is typically greater in energy than food prepared at home [5–7], and contributes to increased mean daily energy intake in both adults and children when eaten at least once a week [8]. A previous study found that among US adults, those who ate fast food or food from full-service restaurants consumed approximately 200kcals more on that day, compared to individuals who ate a meal prepared at home, with additional increases in fat and sodium intake [9].

In the UK, public health strategies to improve the nutritional quality of food sold in the out-of-home food sector have been suggested at national and local levels [10]. In April 2022, regulations were introduced in England requiring calorie labels to be present on all food menus for businesses with over 250 employees, including on food delivery platforms [11, 12]. This policy was implemented with the aim of helping consumers make healthier decisions through reducing the number of calories purchased, despite the evidence of calorie labelling to date being uncertain [13]. A systematic review of calorie labelling research concluded that the impact of labelling is likely specific to the setting in which they are implemented [14]. No research that we are aware of, has considered the impact of calorie labelling on food delivery platforms.

Non-Governmental Organisations have also urged the UK Government to develop policies to address disproportionate pricing of unhealthy foods [15]. Value pricing contributes toward excessive consumption of energy dense, low nutrient foods by attracting consumers to larger (versus smaller) portion sizes for a disproportionately small price difference [16]. An analysis of fast-food combination meals concluded there was a strong financial incentive for price-conscious consumers to size-up [17]. The success of this incentive can be attributed to the consumers’ greater focus on the value of a product compared to other factors such as health [16, 18]. A strategy that could be adopted on food delivery platforms to help discourage the ordering of excess food is proportional pricing whereby consumers are required to pay a standard price per unit (e.g. price per gram) [19], which would increase the price differential between larger and smaller portions (relative to value pricing).

Previous research has assessed the impact of proportional pricing with different foods and beverages in a range of settings and findings are mixed. In a laboratory study, participants were given the option to purchase popcorn with money provided by the researchers [16]. Participants in the value pricing condition were more than two times as likely to purchase a larger size than participants in the proportional pricing condition. However, no effect of proportional pricing was found in a study conducted in a university cinema [20], perhaps due to the relatively small difference in price for the beverage [20].

In a study conducted in the Netherlands [19], fast food customers were asked to complete a questionnaire which involved hypothetically choosing a size of soda and chicken nuggets presented with value pricing or proportional pricing. Proportional pricing reduced the likelihood of choosing the largest size of soft drink and increased the likelihood of choosing the smallest portion size of chicken nuggets in participants with overweight/obesity. Pricing strategies had no effect on the portion size choice of chicken nuggets or soda for participants with normal weight [19].

It is important to consider how implementing a new policy, such as proportional pricing, would interact with the existing food environment. One study conducted in the US examined the influence of a pricing intervention alongside presence or absence of calorie labelling [21], and found that when pricing was regarded as better value, participants were less likely to be influenced by calorie labels. From this, it can be speculated that interventions simultaneously reducing the perceived value of food and increasing the provision of nutritional information, would be the most likely to promote healthier behaviours on food delivery platforms.

Due to the growing popularity of food delivery apps and the need to examine potential interventions to improve dietary choices in this context, the primary aim of the current research was to examine the influence of proportional pricing and calorie labelling interventions, both separately and combined, on portion size choice, calories ordered and monetary value of orders made when selecting food and drinks from three outlet types on a virtual delivery app. Additional aims were to explore whether the effects of proportional pricing and calorie labelling differ according to participant characteristics (i.e. BMI, age, gender, education, ethnicity and food choice motive scores). We hypothesised that there would be a significant impact of pricing strategy, where participants in proportional rather than value pricing conditions would be less likely to choose a larger option, would order fewer calories and spend less money. We also hypothesised that there would be a significant effect of calorie labelling, where participants in calorie labelling conditions would be less likely to choose a larger option, would order fewer calories and would spend less money compared to participants not shown calorie labels. Furthermore, we predicted that the likelihood of choosing smaller options and fewer calories would be most pronounced when participants were provided with both proportional pricing and calorie labels.

## Methods

### Study sample

**Fig. 1:**
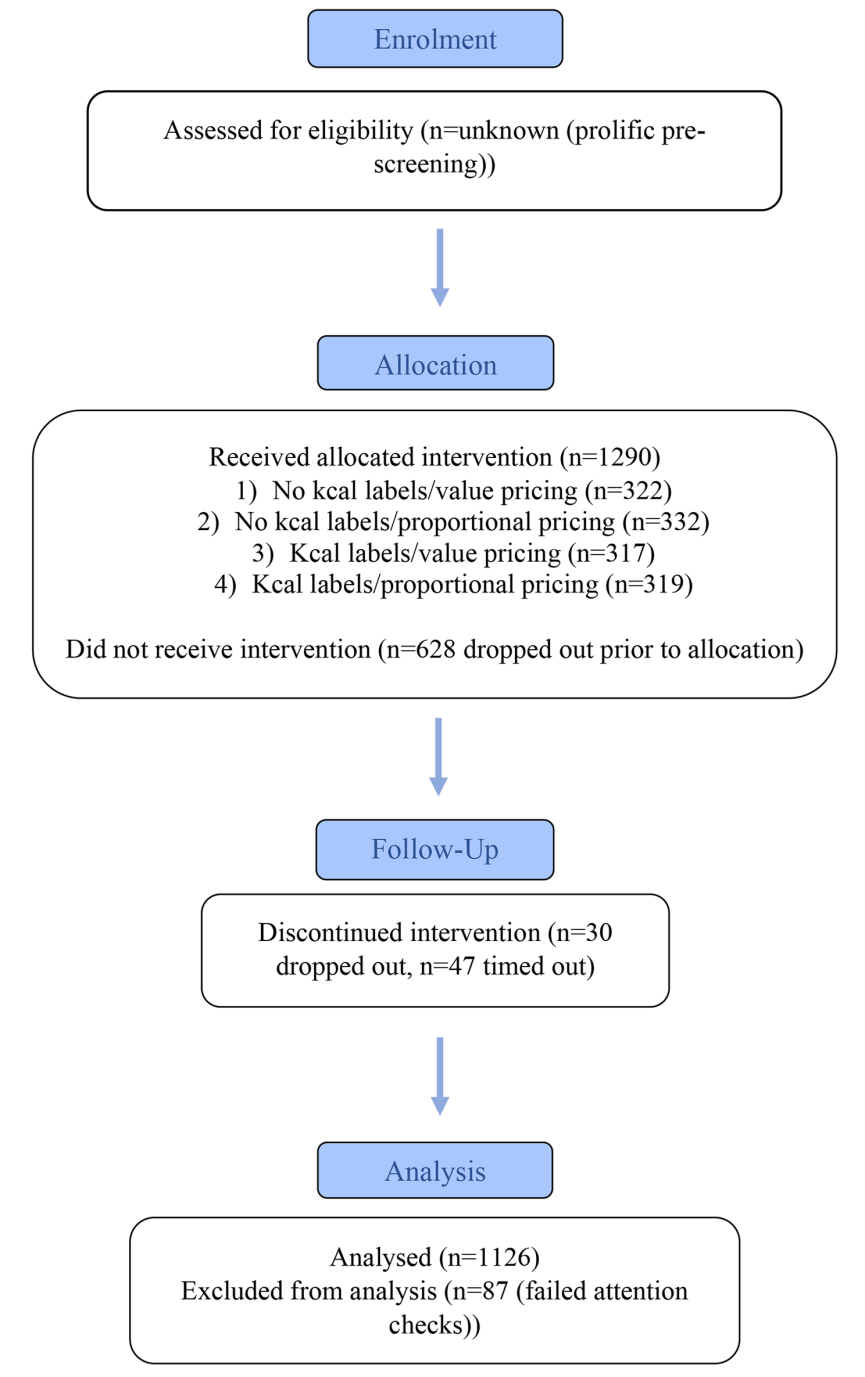
Adapted consort flowchart of participant recruitment through to completion

Participants (final N = 1126) were recruited through the online research platform Prolific [22] between 16th June and 16th August 2022. Participants were eligible to participate in the study if they resided in the UK, were over the age of 18 years, spoke fluent English, frequently used food delivery apps or websites (at least once a month on average), and had access to a laptop or desktop. Participants were ineligible to take part if they were currently pregnant or breastfeeding, partaking in a fast or other restrictive eating practices, had a history of eating disorders, or specific dietary restrictions (vegan, gluten-free, dairy-free, sugar-free). Participants were told this was a study of food ordering, but were not made aware of the study aims and hypotheses. Recruitment was stratified by gender (approx. 50% male, 50% female) and socioeconomic position (SEP; measured by highest level of education) to be representative of the population in the UK (47% National Qualifications Framework (NQF)4+ (degree level or higher), 19% NQF3 (A levels or equivalent), 17% NQF2 (GCSEs or equivalent) and 17% NQF1 (no formal qualifications) [23]. Stratification criteria were obtained through the online research platform Prolific. Further recruitment details are in supplementary material I). If participants completed the study, they received monetary reimbursement (equivalent to £7-£9/hour). Figure 1 presents a CONSORT flow diagram of study participation.

### Design

This study used a 2 × 2 between-subjects design. Upon recruitment, participants were randomly assigned to one of four experimental conditions: calorie labels and value pricing; calorie labels and proportional pricing; no calorie labels and value pricing; no calorie labels and proportional pricing. Participants were recruited via the online platform Prolific before being provided with a web link to complete all aspects of the study procedure on Inquisit 6. Randomisation with 1:1:1:1 allocation was performed using the ‘<batch>’ and ‘/subjects’ functions in Inquisit 6.

### Demographic and participant characteristic measures

Online questionnaires collected self-reported data on gender, age, ethnicity, height, weight, and SEP. The primary measure of SEP was the highest educational level achieved, for which there were six options (less than high school, high school completion, college or foundation degree, bachelor’s degree, master’s degree, and doctoral or professional degree). Additional SEP measures collected were subjective social status and equivalised household income. For subjective social status, participants completed the MacArthur Scale of Subjective Social Status (SSS) [24], where they were required to rank themselves on a ladder (rating of 1–10) symbolising where they stood in society. The bottom rung of the ladder was labelled 1: ‘representing those with the least money, least education and worst jobs or no jobs’ and the top rung was labelled 10: ‘representing those who have the most money, most education and best jobs’. Participants reported their after-tax household income (to the nearest £1000) and the number of adults and children residing in their household. Values were applied to household members (first adult = 1, additional adult or child over 14 years = 0.5, child aged 0–13 = 0.3) and equivalised household income was calculated by dividing the household income by the value of household members [25].

### Food choice motives

After completing orders from all three food outlets (described below), participants were presented with subscales of the Food Choice Motives Questionnaire [26]. Five food choice motives deemed relevant potential impacts of calorie labelling and pricing (health, sensory appeal, price, weight control and familiarity) were included. Questionnaire items (e.g. “It is important to me that the food I eat on a typical day is good value for money”), were rated on a scale of [1] ‘not important at all’ to [4] ‘very important’. Each food choice motive consisted of 3–5 items. Sub-scale scores were the sum of respective items.

### Virtual delivery app

A virtual delivery app (Supplementary material II) was created using the program Inquisit 6, and modelled on a popular UK-based food delivery platform. Three large chain food outlets in the UK (a coffee shop, a sandwich shop, and a fast food outlet) were simulated as they each typically offer products at a larger size with value pricing, and cover a range of food types ordered through delivery apps (i.e., beverages, sandwiches, meals). After completing baseline questionnaires, participants were informed they would be completing hypothetical food and drink orders, and asked to imagine they were going to pay for and receive the items ordered. On the following page, participants were asked to imagine they were ordering a drink from a coffee shop chain at whichever time of day they would usually purchase a drink from a café. There were 21 possible beverages for participants to order. Once participants had chosen their drink, they were asked to select a size (medium or large, except for n = 4 beverages where only a medium was available). Finally, participants specified addition of milk, sugar or sweetener, and other preferences.

For the sandwich outlet order, participants were asked to imagine they were ordering a sandwich for lunch. There were 21 possible filling options. Once a choice was made, participants were required to select a size (6-inch or 12-inch) and then make various specifications (bread type, additional fillings, sauce). Participants were then asked if they wanted to see the sides menu. If they selected yes, they were shown the 15 available sides which they could add to their order or continue, before being asked if they wanted to see the drinks menu. If they selected yes, participants were shown the 15 available drinks which they could add to their order or complete their order.

**Fig. 2:**
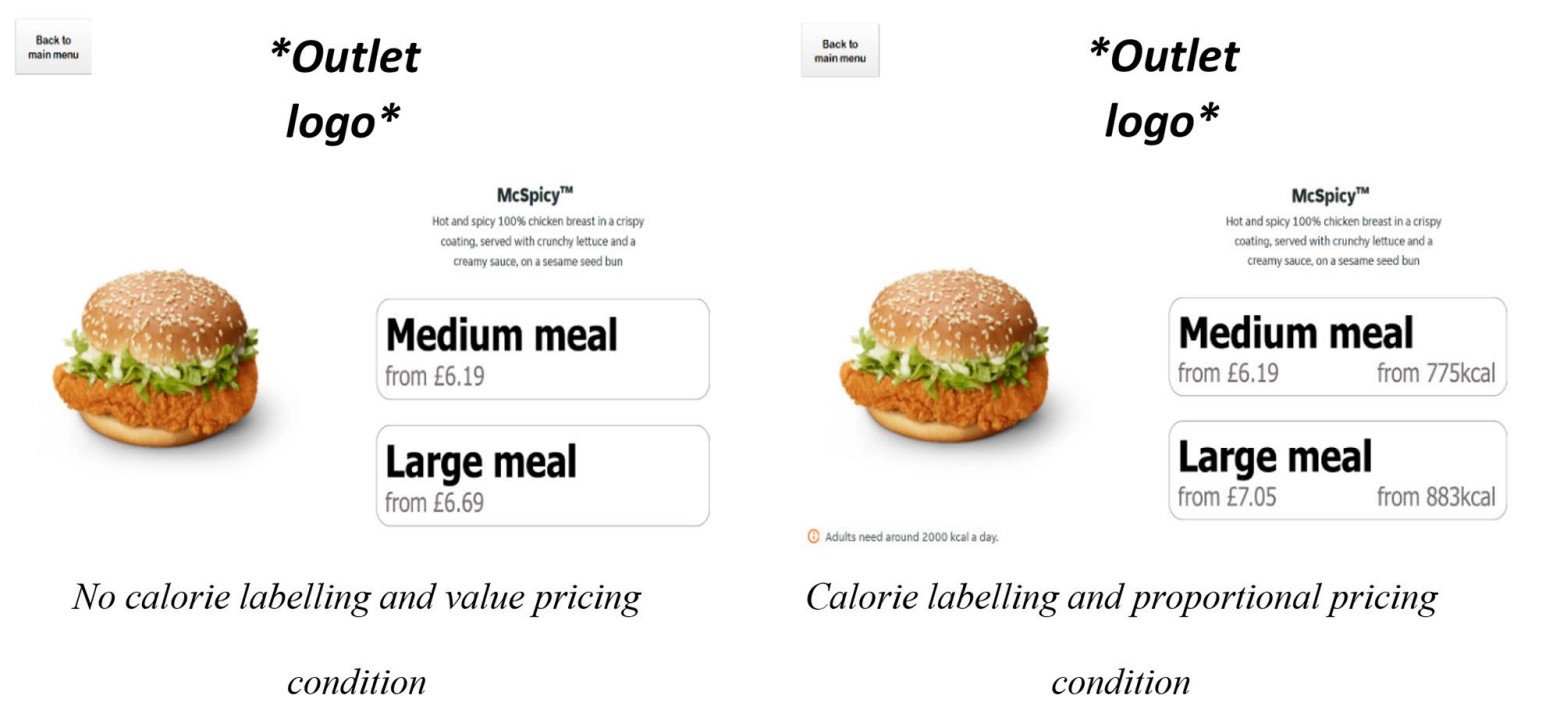
Example of virtual delivery app

For the fast food outlet, participants were asked to imagine they were ordering an evening meal. There were 21 possible main meal component options (e.g. burgers, wraps, chicken dishes). Once participants made their choice, they were asked to specify a meal size (medium or large; for an example screen shot see Fig. 2.), and select a side (n = 4 options) and a beverage (n = 22 options). Following this, participants were asked if they would like to see the additional sides menu. If they selected ‘yes’ participants were shown the 15 additional sides which they could add to their order or complete their order.

Prices, calorie content and presentation of menu items were obtained in 2022 from a major UK food delivery platform. In calorie labelling conditions, each food option had calorie information in the description, and a statement of calorie guidelines visible on the page (“Adults need around 2000kcals a day”). For proportional pricing conditions, prices were calculated for any items with multiple size options. Prices for larger sizes were determined by calculating the percentage increase in kilocalories (herein: kcals) between the smaller and larger size options and translating that into the same percentage increase in price (see supplementary material III for an example).

### Other measures

After providing consent, participants were asked to guess the aim of the study. Guesses relating to kcals/calorie information on food choice or food price on food choice were coded as correctly guessed. Aim guessing was coded independently by two authors (AF, RM) and inconsistencies were resolved through discussion or consultation with another author (ER) where necessary (n = 8). Finally, participants were asked questions relating to the representativeness of the virtual app, the typicality of their orders, whether they believed they were influenced by the calorie content or the price of the foods, whether they supported calorie labelling and proportional pricing interventions in the out-of-home food sector, and if they believed either intervention would be successful in helping people to make healthier choices. Throughout the course of the study, there were three attention checks. If any participant failed one of the three attention checks (e.g. How many times have you visited the planet Mars?), their data were excluded from all analyses.

### Procedure

After providing consent, participants were randomly assigned to one of the four conditions. Baseline demographic data were collected, and participants completed items on how frequently food apps generally, and the three simulated food outlets in the study, were used by participants. Participants next completed hunger and thirst ratings and following this, took part in the food ordering task for all three outlets (order as above) on the virtual delivery app. A final questionnaire measured food choice motives and additional measures. All questions are shown in Supplementary material IV. Finally, participants were fully debriefed. Participant completed the full study once, and on average, the experiment took approximately 12 min to complete. Data collection took place from the 2nd to the 17th August 2022.

### Analysis

We followed a pre-registered analysis strategy (osf.io/kaju5 [27]). For minor deviations see Supplementary Material V. Data from the three outlets were analysed separately in planned primary analyses. ‘R’ was used to conduct analyses with the following packages: ‘descr’ [28] using function ‘freq’; performance’ [29] with functions ‘check_distribution’, ‘check heteroscedasticity’ and ‘check_model’; ‘stats’ [30] with function ‘glm’; and ‘estimatr’ [31] with function ‘lm_robust’.

#### Primary analyses

Primary outcomes for this study were size choice (medium or large), hypothetical kcals ordered, and hypothetical money spent from each outlet. For each outlet, binary logistic regressions were used to examine the association between calorie labelling condition (present/absent) and pricing condition (proportional/value) as predictors of product size choice. Body Mass Index (BMI), highest level of education (university educated or less than university educated), the five food choice motives, age, gender (male/non-male) and ethnicity (white/non-white) were included as covariates. Multiple linear regressions with robust standard errors (and the same predictors as mentioned above) were used to examine associations between pricing condition and calorie labelling condition with total kcals ordered and total money spent. Results for primary analyses were considered significant at p < .05. For all models we examined the impact of including the interaction between labelling condition and pricing condition as a predictor. All interactions were non-significant, so these were removed from the separate models (i.e. size choice, kcals ordered, hypothetical spend) to minimize model overfitting.

#### Unplanned additional analyses

Data from the three outlets were combined to examine any overall effects of calorie labelling condition and proportional pricing condition across the three outlet types. For size choice, participants were given a score of 0 to 3 according to the number of times they selected a larger portion size. A linear regression model was used to examine associations of calorie labelling condition, pricing condition and their interaction with this outcome. For both kcals ordered and hypothetical spend, mixed ANOVAs were used to observe any main effects of calorie labelling condition, pricing condition and their interaction on the outcomes. In the mixed ANOVAs, the within-subjects factor was the outlet and the between-subjects factors were calorie labelling condition and pricing condition.

#### Secondary analyses

Interactions between participant characteristics (BMI, education, food choice motives, age, gender and ethnicity) and experimental conditions were investigated at a second step in each of the models outlined in the primary analyses. Results for secondary analyses were considered significant at p < .01 to account for multiple testing.

#### Sensitivity analyses

Sensitivity analyses (Supplementary Material VI) revealed that results were largely the same whether subjective social status or equivalised household income were used in place of highest education level as a measure of SEP. Additional sensitivity analyses identified no differences in significance for primary findings when participants with a missing BMI (n = 6) were removed (analyses not reported). Changes to primary findings when participants who guessed the aim of the study were removed (n = 98) are reported in the results section.

#### Sample size calculation

G* Power was used to conduct an a-priori sample size calculation. Based on previous research [21, 32], we powered the study (See supplementary material VII for full information) to detect medium effect sizes (f = 0.24) of proportional pricing, calorie labelling and interaction effects at 0.80% power with an error probability of 0.05. We aimed to recruit a sample of N = 1200 participants.

## Results

Due to participant drop-out and failed attention checks, N = 1126 participants (94% of intended sample) were included in the final sample for analyses. See Fig. 1 for a flowchart of participant recruitment through to completion. Participants had a mean age of 40.21 years (SD = 13.6), the majority were White (86%), 50% were university educated, and 49% were female. Participant characteristics overall and by condition are reported in Table 1. Overall, our sample was broadly representative of the socio-demographic breakdown of the UK.

**Table 1:** Participant characteristics overall, and across the four conditions

Categories	Overall (n = 1126)	No kcal labels/value pricing) (n = 277)	No kcal labels/ proportional pricing) (n = 294)	Kcal labels/ value pricing) (n = 270)	Kcal labels/ proportional pricing) (n = 285)
**Gender**					
Male	563 (50%)	143 (52%)	147 (50%)	138 (51%)	135 (47%)
Female	557 (49%)	130 (47%)	146 (50%)	131 (49%)	150 (53%)
Other	6 (< 1%)	4 (1%)	1 (< 1%)	1 (< 1%)	0 (0%)
**Ethnicity**					
White	969 (86%)	242 (87%)	254 (86%)	223 (83%)	250 (88%)
Non-white	157 (14%)	35 (13%)	40 (14%)	47 (17%)	35 (12%)
**Education**					
Less than university educated	564 (50%)	141 (51%)	157 (53%)	128 (47%)	138 (48%)
University educated	562 (50%)	136 (49%)	137 (47%)	142 (53%)	147 (52%)
**Age**					
Mean	40.21	39.97	39.26	40.32	41.34
SD	13.6	13.64	13.69	12.87	14.11
Range	18–80	18–80	18–76	19–76	18–78
**BMI**					
Mean	27.00	26.95	27.04	27.39	26.65
SD	6.19	6.26	6.15	6.62	5.74
Range	14.37–59.23	14.45–55.56	16.02–55.02	16.64–59.23	14.37–51.96
Missing/ implausible	N = 6	N = 3	N = 1	N = 2	N = 0

Categorical variables are represented by counts/percentages, and continuous variables by means/standard deviations.

### Primary analyses

#### Food orders

**Fig. 3:**
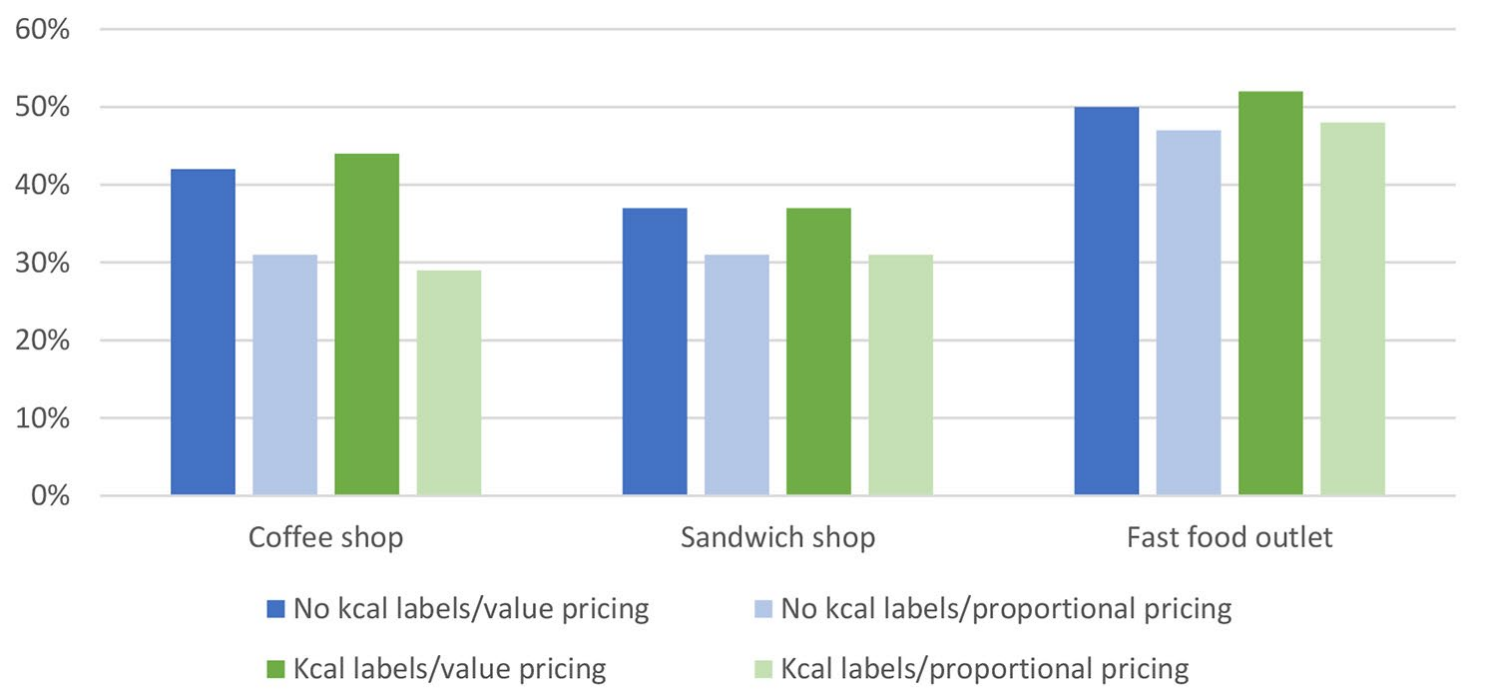
The proportion of participants opting for a larger size option for all three outlets across the four conditions

**Fig. 4:**
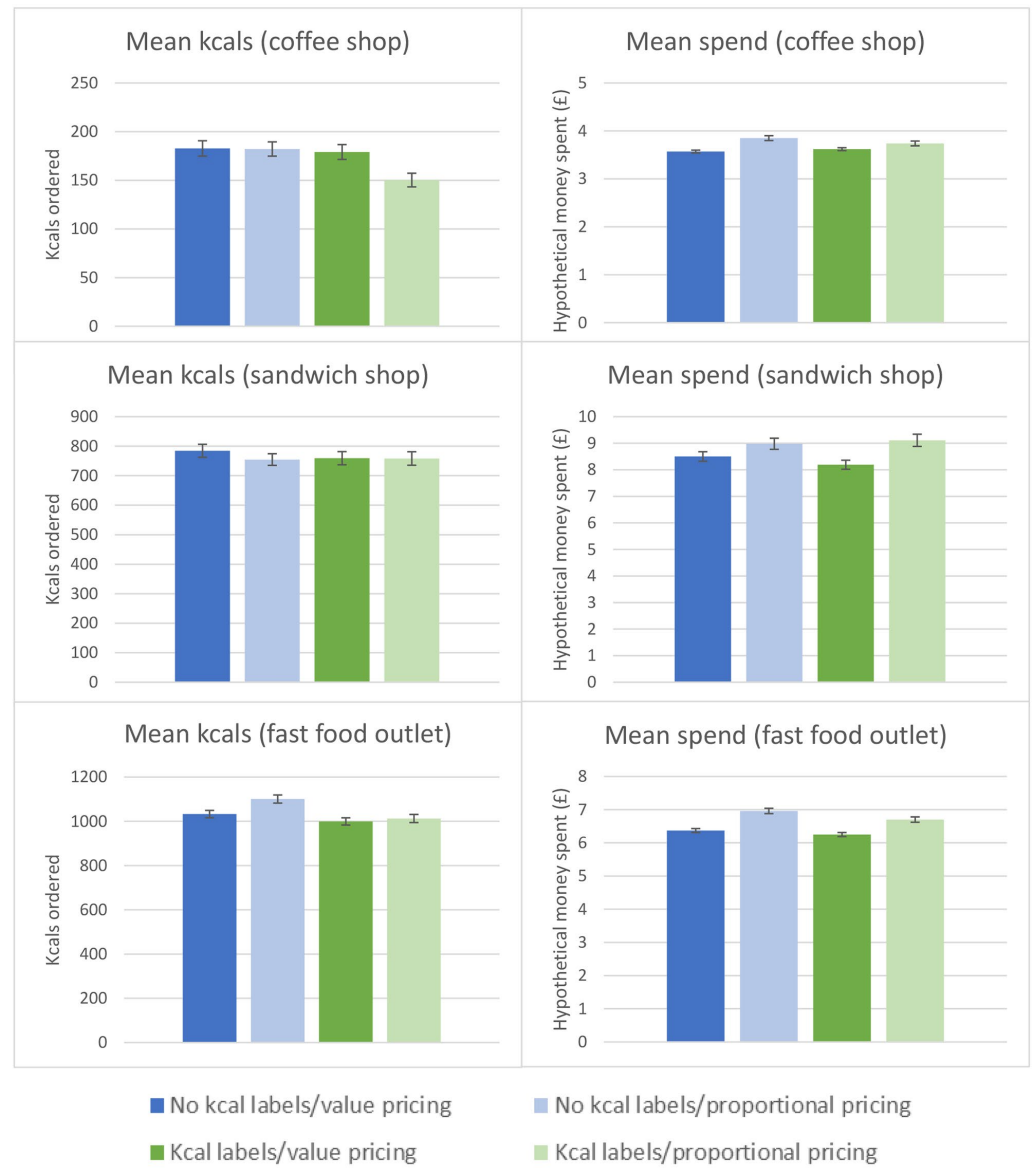
Mean kcals ordered and hypothetical money spent from the coffee shop, sandwich shop and fast food outlet across the four conditions. Error bars represent the standard error

Mean orders for the four conditions are presented fully in Supplementary material VIII. Data of size choice are visually represented in Fig. 3, and mean kcals ordered and hypothetical spend are visually represented in Fig. 4.

#### Impact of calorie labelling

There were no main effects of calorie labelling on size choice, consistent across all three food outlets (Table 2). There was some evidence that calorie labelling reduced kcals ordered. When participants were presented with calorie labels, there was an observed decreased purchase of kcals for both the coffee shop (-18.95kcals, 95% CI -33.07 to -4.84) and the fast food outlet (-54.19kcals, 95% CI -86.04 to -22.33) but not the sandwich shop. There were inconsistent findings of calorie labelling on hypothetical spend. For both the coffee shop and sandwich shop, there were no main effects of calorie labelling condition on hypothetical money spent, however being presented with calorie labels was associated with decreased spend from the fast food outlet (-£0.17, 95% CI -0.30 to -0.03).

When data from the three outlets were combined, there was no main effect of calorie labelling on product size choice (F(2,1123) = 5.84, p = .717, adjusted R^2^ = 0.01) or hypothetical spend (F(1,1121) = 1.44, p = .230, ηp^2^ < 0.01). A significant main effect of calorie labelling condition on kcals ordered was observed (F(1,1121) = 6.11, p = .014, ηp^2^ (generalised) < 0.01), whereby participants in labelling conditions ordered 29 fewer calories overall than those in the non-labelling conditions. Findings were similar using linear models with robust standard errors to account for heteroscedasticity.

**Table 2:** Regression analyses for calorie labelling on size choice, kcals ordered and money spent for the three food outlets

	*Estimate*	*Std. error*	*Sig.*	*Odds ratio*	*95% CI* *Lower*	*95% CI Upper*
*Size choice* ^*1*^						
Coffee shop beverage size						
*Pseudo R* ^*2*^	0.067					
*Labelling condition (kcal labels)*	0.03	0.13	0.843	1.03	0.80	1.32
Sandwich size						
*Pseudo R* ^*2*^	0.156					
*Labelling condition (kcal labels)*	0.09	0.14	0.509	1.09	0.83	1.43
Fast food meal size						
*Pseudo R* ^*2*^	0.124					
*Labelling condition (kcal labels)*	0.11	0.13	0.376	1.12	0.87	1.44
*Kcals ordered* ^*2*^						
Coffee shop						
*Adjusted R* ^*2*^	0.097					
*Labelling condition (kcal labels)*	**-18.95**	**7.19**	**0.009**	-	**-33.07**	**-4.84**
Sandwich shop						
*Adjusted R* ^*2*^	0.119					
*Labelling condition (kcal labels)*	-0.43	20.37	0.983	-	-40.39	39.53
Fast food outlet						
*Adjusted R* ^*2*^	0.178					
*Labelling condition (kcal labels)*	**-54.19**	**16.24**	**< 0.001**	-	**-86.04**	**-22.33**
*Money spent* ^*3*^						
Coffee shop						
*Adjusted R* ^*2*^	0.082					
*Labelling condition (kcal labels)*	-0.02	0.04	0.575	-	-0.10	0.06
Sandwich shop						
*Adjusted R* ^*2*^	0.112					
*Labelling condition (kcal labels)*	-0.02	0.19	0.924	-	-0.39	0.36
Fast food outlet						
*Adjusted R* ^*2*^	0.138					
*Labelling condition (kcal labels)*	**-0.17**	**0.07**	**0.016**	-	**-0.30**	**-0.03**

^1^Estimates indicate the average change in the log odds of the response variable associated with a one unit increase in each predictor variable, ^2^ Estimates are in kcals, ^3^Estimates in pound sterling

#### Impact of proportional pricing

There were inconsistent effects of pricing condition on portion size choice (Table 3). Being in the proportional pricing condition was associated with a decreased likelihood of choosing a larger sized beverage from the coffee shop (OR = 0.58, 95% CI 0.45 to 0.75). For both the sandwich shop and fast food outlet, there were no significant main effects of pricing on size choice. Similarly, inconsistencies were observed for effects of pricing condition on kcals ordered, whereby no significant main effects of pricing on kcals ordered were observed for orders from the coffee shop or sandwich shop. However, being presented with proportional pricing was associated with an increase in kcals ordered from the fast food outlet (51.25kcals, 95% CI 19.59 to 82.90). Pricing condition was significantly associated with hypothetical spend for all three outlets whereby being in the proportional pricing condition was associated with increased money spent in the coffee shop (£0.21, 95% CI 0.13 to 0.29), sandwich shop (£0.78, 95% CI 0.41 to 1.16) and the fast food outlet (£0.57, 95% CI 0.43 to 0.70).

When data from the three outlets were combined, a main effect was observed of pricing condition on size choice (F(1,1123) = 5.84, p < .001, adjusted R^2^ = 0.01) whereby participants chose a larger size 0.2 times less frequently in the proportional pricing condition (mean times larger size chosen = 1.09 times) compared to the value pricing condition (mean times larger size chosen = 1.31 times). There was no significant main effect of pricing condition on calories ordered (F(1,1121) = 0.06, p = .808, ηp^2^(generalised) < 0.01) but there was a main effect of pricing condition on hypothetical spend (F(1,1121) = 31.05, p < .001, ηp^2^(generalised) = 0.01) whereby participants in the proportional pricing conditions spent £0.47 more than participants in the value pricing conditions. Findings were similar using linear models with robust standard errors.

**Table 3:** Regression analyses for proportional pricing on size choice, kcals ordered and money spent for the three food outlets

	Estimate			Std. error		Sig.	Odds ratio	95% CI Lower		95% CI Upper
*Size choice* ^*1*^										
Coffee shop beverage size										
*Pseudo R* ^*2*^		0.067								
*Pricing condition (proportional)*		**-0.54**	**0.13**		**< 0.001**		**0.58**		**0.45**	**0.75**
Sandwich size										
*Pseudo R* ^*2*^		0.156								
*Pricing condition (proportional)*		-0.23	0.14		0.098		0.79		0.60	1.04
Fast food meal size										
*Pseudo R* ^*2*^		0.124								
*Pricing condition (proportional)*		-0.07	0.13		0.584		0.93		0.72	1.20
*Kcals ordered* ^*2*^										
Coffee shop										
*Adjusted R* ^*2*^		0.097								
*Pricing condition (proportional)*		-13.75	7.26		0.058				-27.99	0.48
Sandwich shop										
*Adjusted R* ^*2*^		0.119								
*Pricing condition (proportional)*		-1.77	20.62		0.932				-42.23	38.70
Fast food outlet										
*Adjusted R* ^*2*^		0.178								
*Pricing condition (proportional)*		**51.25**	**16.13**		**0.002**				**19.59**	**82.90**
Money spent^3^										
Coffee shop spend										
*Adjusted R* ^*2*^		0.082								
*Pricing condition (proportional)*		**0.21**	**0.04**		**< 0.001**				**0.13**	**0.29**
Sandwich shop spend										
*Adjusted R* ^*2*^		0.112								
*Pricing condition (proportional)*		**0.78**	**0.19**		**< 0.001**				**0.41**	**1.16**
Fast food outlet spend										
*Adjusted R* ^*2*^		0.138								
*Pricing condition (proportional)*		**0.57**	**0.07**		**< 0.001**				**0.43**	**0.70**

^1^Estimates indicate the average change in the log odds of the response variable associated with a one unit increase in each predictor variable, ^2^ Estimates are in kcals, ^3^ Estimates in pound sterling

Sensitivity analyses revealed that when aim guessers were removed, being in the proportional pricing conditions as opposed to value pricing conditions was associated with a decreased likelihood of choosing a larger sized sandwich from the sandwich shop (OR = 0.74 95% CI 0.56 to 0.98). When equivalised household income was included in the regression model instead of educational level, being presented with proportional pricing was significantly associated with reduced kcals ordered from the coffee shop (-14.68kcals, 95% CI -28.85 to -0.50).

Interaction between calorie labelling and proportional pricing.

There were no significant interactions between calorie labelling and pricing strategy on size choice, kcals ordered, or hypothetical money spent from any outlet (Table 4). Additionally, when data from the three outlets were combined, there were no significant interactions between calorie labelling condition and pricing condition on size choice (F(3,1122) = 3.93, p = .662, Adjusted R^2^ = 0.01), calories ordered (F(1,1121) = 0.67, p = .413, ηp^2^ < 0.01) and hypothetical spend (F(1,1121) = 0.09, p = .762, ηp^2^ < 0.01). Findings were similar using linear models with robust standard errors.

#### Secondary analyses

Secondary analyses (supplementary material IX and X) revealed a significant interaction (29.57kcals, 99% CI 4.53 to 54.60) between calorie condition and weight control food choice motives on kcals ordered from the coffee shop. However, when broken down, associations between weight control and kcals ordered were not significant in labelling or non-labelling conditions. A small significant interaction was also observed between proportional pricing and BMI on hypothetical spend from the coffee shop (£0.02, 99% CI 0.00 to 0.04) and sandwich shop (£0.10, 99% CI 0.03 to 0.17), whereby having a higher BMI was associated with a greater spend in proportional pricing vs. value pricing conditions, but not for fast food orders. Across all three food outlets, education level, ethnicity, gender, and age, as well as price, health, sensory and familiarity food choice motives did not significantly interact with calorie labelling or proportional pricing on the outcomes of interest.

**Table 4:** Regression analyses for interactions between calorie labelling and proportional pricing on size choice, kcals ordered, and money spent for the three food outlets

	*Estimate*	*Std. error*	*Sig.*	*Odds ratio*	*95% CI* *Lower*	*95% CI Upper*
*Size choice* ^*1*^						
Coffee shop beverage size						
*Pseudo R* ^*2*^	0.067					
Kcal labels*proportional pricing	-0.14	0.25	0.588	0.87	0.52	1.44
Sandwich size						
*Pseudo R* ^*2*^	0.156					
Kcal labels*proportional pricing	0.15	0.28	0.590	1.16	0.68	1.99
Fast food meal size						
*Pseudo R* ^*2*^	0.124					
Kcal labels*proportional pricing	0.08	0.26	0.296	1.08	0.65	1.79
*Kcals ordered* ^*2*^						
Coffee shop						
*Adjusted R* ^*2*^	0.097					
Kcal labels*proportional pricing	-19.75	14.46	0.172	-	-48.12	8.61
Sandwich shop						
*Adjusted R* ^*2*^	0.120					
Kcal labels*proportional pricing	48.67	41.51	0.241	-	-32.77	130.11
Fast food outlet						
*Adjusted R* ^*2*^	0.179					
Kcal labels*proportional pricing	-42.91	32.17	0.183	-	-106.04	20.21
*Money spent* ^*3*^						
Coffee shop						
*Adjusted R* ^*2*^	0.083					
Kcal labels*proportional pricing	-0.11	0.08	0.175	-	-0.27	0.05
Sandwich shop						
*Adjusted R* ^*2*^	0.114					
Kcal labels*proportional pricing	0.62	0.38	0.104	-	-0.13	1.38
Fast food outlet						
*Adjusted R* ^*2*^	0.138					
Kcal labels*proportional pricing	-0.11	0.14	0.417	-	-0.38	0.16

^1^ Estimates indicate the average change in the log odds of the response variable associated with a one unit increase in each predictor variable, ^2^ Estimates are in kcals, ^3^ Estimates in pound sterling

### Exploratory analyses

Exploratory analyses were conducted to investigate the source of the unexpected increased kcals ordered from the fast food outlet in proportional pricing conditions (Supplementary material XI). There was no main effect of pricing condition on kcals ordered from meals ordered, but there was for kcals from additional optional side dish orders; being in the proportional pricing condition was associated with increased kcals ordered from additional sides (54.44kcals, 95% CI 37.94 to 70.93).

### Questionnaire responses

The majority (> 75%) of participants agreed that the virtual delivery app was representative of existing food delivery apps, and reported their orders where typical of what they would normally order. 20% of participants agreed that the food choices they made were influenced by how many calories they thought were in the food options and 33% agreed they were influenced by price. Over 60% of participants agreed with the use of calorie labelling and proportional pricing policies in the out of home food sector and tended to believe these policies would help people to make healthier choices (See online supplementary Material IV),

## Discussion

This study examined the impact of calorie labelling and proportional pricing in a virtual online food delivery platform on portion size choice, kcals ordered and hypothetical spend from beverage, sandwich and fast food outlets. Compared to non-labelling conditions, calorie labelling had no significant impact on portion size selection of food or beverages from the coffee shop, sandwich shop, fast food outlet or when data were combined across all three outlet types. Calorie labelling did however significantly reduce total energy ordered in the coffee shop (-18.95kcals) and fast food outlet (-54.19kcals), but not significantly from the sandwich outlet (-0.43kcals). When data across outlets were combined, there was a small overall main effect of calorie labelling on energy ordered. For the fast food outlet only, calorie labelling was associated with a reduced total spend and in pooled analyses across the three outlets there was no difference in total spend based on calorie labelling condition. Presenting food and beverage items with proportional pricing (as opposed to standard value pricing) was associated with a decreased likelihood of choosing a larger beverage size from the coffee shop outlet by 42%. This statistically significant decrease was not found for the sandwich shop (21% reduction) or fast food outlet (7% reduction). However, when data were combined across outlets, there was an overall effect of proportional pricing, whereby presence of proportional pricing was associated with the participant selecting the larger portion size 0.2 times less (out of a possible 0–3 times) on average across the three outlets (a 7% reduction in number of times large portion size was chosen). Unexpectedly participants in the proportional pricing condition had a significantly higher total energy content of orders for the fast food outlet, as opposed to small non-significant decreases in the other two outlets. In all three outlets, proportional pricing was associated with increased hypothetical spend, and likewise a main effect of pricing condition was observed for combined outlets. No consistent interactions between a range of participant characteristics and interventions were identified across the different outlet types. Nor did we find any evidence that the effect of calorie labelling differed as a function of pricing condition for any of the outcomes.

There was no evidence that calorie labelling had an effect on portion size selection of food or beverages across any outlet, but consistent with some research examining hypothetical food choice, labelling was associated with reductions in energy ordered [14, 32]. It may be that calorie labelling has a greater influence on consumer choice through the type of products and/or number of products selected as opposed to product portion size selections. However, there was no evidence that orders from the sandwich shop outlet were significantly affected by calorie labelling. In a previous study conducted in the US [33], calories purchased in the same sandwich outlet increased following implementation of calorie labelling, while this decreased or stayed the same for other outlets. Research designed to understand whether (and if so, why) effects of calorie labelling differ by outlet type would be informative.

The majority of participants were supportive of calorie label interventions in the out-of-home food sector, which is consistent with findings from others studies [34]. There has been some negative responses to the introduction of these labels in the UK [35, 36], largely fuelled by the potential impacts on individuals with eating disorders. Previous research has identified no causal adverse effects of labelling on individuals with a high risk of eating pathologies, including changes to emotional state and unhealthy behaviours [37] and evidence suggests that labelling policies have the greatest public support when compared with alterations to product size, availability or tax [38, 39]. However, the impacts of this kind of policy on individuals with eating disorders should continue to be explored.

In proportional pricing conditions vs. standard value pricing, when data across outlets were combined, participants overall selected a larger portion size less frequently. For the separate outlets, this relationship was only significant for beverage purchases from the coffee shop. The same trends were observed for the sandwich shop (-21%) and fast food outlet (-7%) although differences were not statistically significant. It may be that a larger impact would be observed if a smaller size was offered for a proportionately lower price. In this study, participants were only able to choose between medium and large portion sizes. Previous research has found that increasing the number of options at the larger end of the scale (i.e., adding an extra-large option to small, medium, large) increased the number of people opting for the large size [40]. This was explained by extremeness aversion, whereby consumers tend to avoid extreme options and choose a middle option. However, this effect was not observed when calorie labels were present, as the authors speculate that consumers become warier of the health costs. The addition of a smaller size option for proportionally less money alongside calorie labelling could have the greatest impact in regard to nudging consumers toward smaller sizes. Future research should consider this, as there would be potential for health and monetary benefits for consumers.

Proportional pricing was associated with small non-significant decreases in total energy ordered in the coffee shop and sandwich outlets, but an unexpected increase in the fast food outlet. Exploratory analyses revealed that proportional pricing was associated with increased ordering of optional additional side dishes. In the study, proportional pricing was not presented for all side dishes (due to there being only one size) and many of the additional sides were low in price. Therefore, the effect of proportional pricing on total energy ordered may reflect lower priced items being perceived as better value for money when relative prices of larger meals increased (due to proportional pricing). Previous research has shown that many people rate the financial value of food products over factors such as nutritional content [16]. Therefore, for proportional pricing to be effective in reducing energy content of orders, this pricing strategy may need to be applied consistently across menus and in concert with pricing strategies to address single size lower price and higher energy menu options.

In all three food outlets, proportional pricing was significantly associated with increased spend, showing that larger options are still attractive to many consumers even when more expensive. Although participants were broadly in support of proportional pricing (> 60%), across all three outlets, 90% of participants said that orders were typical for them (in terms of content and size). The lack of consistent effects of proportional pricing in the present study indicate that other complementary approaches, such as reformulation of existing food products, will be valuable in the out of home food sector [41].

In the present study, we found a small increased spend in individuals with a greater BMI in proportional pricing conditions (relative to value pricing) for two of the three outlets, but no interactions with kcals ordered. There was also a small amount of inconsistent evidence that calorie labelling may exert a slightly stronger effect on kcals ordered among participants higher in weight control motives. Given the overall lack of convincing evidence across outcomes for moderation of calorie labelling or proportional pricing on outcomes by a range of participant level characteristics (including indicators of SEP) effects of these interventions may be largely similar across different sociodemographic groups. It is important to consider the role of habitual behaviour and preference in food purchasing. Specifically, it would be interesting to examine whether interventions such as those tested in the present study can override habitual behaviour and existing preferences, or whether intervention effects are limited to non-habitual choice and purchasing behaviours. Research suggests that when a behaviour is habitual, little information is required to make decisions, and individuals pay little attention to alternatives [42]. Additionally, evidence suggests that to override a habit requires conscious behaviour [43]. In the present study, individuals may have been less likely to pay attention to calorie labels or the price of items if they habitually purchased from one of the three outlets. Those who were consciously attending to the calorie labels and prices however, may have been able to override habitual purchasing behaviour. While we collected data on how typical orders were for participants, future research would benefit from exploring whether interventions can cause long-term changes to habitual choice and purchasing behaviour.

### Strengths and limitations

This study was pre-registered and was stratified to represent the UK population in terms of gender and education. While virtual methodologies for food ordering are widely used [44, 45], and predictive of real-world behaviour [46], they lack ecological validity, as participants do not actually pay for or receive the meals. Although, the majority of participants agreed that the virtual app was representative of existing food delivery apps, and that orders from the three outlets were typical for them, the hypothetical nature of behaviour examined in the present study is a limitation. For example, it may be the case that pricing interventions exert a larger influence on behaviour when participants are required to spend their own money.

As is standard practice in eating behaviour research, individuals who were currently dieting, fasting, or had a history of eating disorders we ineligible to take part in this study, and so our findings are not generalisable to these groups. Some previous research has found interactions of calorie labelling with ethnicity [47], whereby some minority groups reported using calorie labels more than their white counterparts. The present study sampled predominantly white participants (in line with UK demographics) and therefore replication of findings in more ethnically diverse groups would now be valuable.

For two of the three outlets, participants had the option to order additional sides, but for the third outlet (a coffee shop) participants were instructed to only order a drink to ensure participant’s focus was on beverage choice per se, rather than considering the beverage as a side option to a food purchase. As we found some evidence in one of the outlets that proportional pricing was associated with increased purchase of side dishes, it is unclear if the same pattern of results would have been observed in the coffee shop outlet. It may be beneficial for future research to consistently include the option of additional sides to be better placed to examine intervention effects on overall diet. Due to the design of the delivery app, participants were required to complete the study on a computer or laptop, but food delivery orders are often made through mobile phones applications. This is shown by the large number of mobile downloads of such apps, for example the UberEats app was downloaded by 3.5 million people in the UK in 2022 alone [48]. In addition, we did not randomise the order of outlets participants were presented with and this may have introduced unintended order effects.

Only three outlet types were examined, all of which were large chains. A wider range of food and beverage outlets are available on food delivery apps. It would be particularly beneficial to know whether orders from smaller non-chain businesses (currently exempt from calorie labelling laws in the US and UK [12]) would be impacted in the same way as observed in the present study, to inform future decision making around whether these types of outlets should also be mandated to provide menu calorie information. Finally, we corrected for multiple comparisons in secondary but not primary analyses. However, with the exception of two findings (calorie labelling on fast food spend; calorie labelling on kcals ordered from combined outlets), primary findings were significant at p < .01 and so little change to our main findings would be observed were further adjustments to the significance value made.

## Conclusions

Calorie labelling on food delivery platforms may effectively reduce calories ordered. Proportional pricing may prompt consumers to select smaller portion sizes, although further research in real-world settings will now be valuable.

### Electronic supplementary material

Below is the link to the electronic supplementary material.

Supplementary Material: TIDieR Checklist

Supplementary Materials I-XI

## Data Availability

The data that supports these findings will be available from the Open Science Framework repository upon publication (osf.io/kaju5 [27]).

## Abbreviations

kcals: Kilocalories
SEP: Socioeconomic position
BMI: Body Mass Index
SSS: Subjective Social Status
