# Supplementary material for: The impact of calorie labelling and proportional pricing on out of home food orders: a randomised controlled trial study using a virtual food and drink delivery app
Source: Int J Behav Nutr Phys Act. 2023 Sep 19;20:112. doi: 10.1186/s12966-023-01513-2 (PMC10508026; doi:10.1186/s12966-023-01513-2)
Supplement: Supplementary file 2 — Supplementary Materials I-XI [file 12966_2023_1513_MOESM2_ESM.docx]

# Supplementary materials

## Supplementary material i: Details of recruitment through Prolific

The study was advertised on Prolific to eight demographic groups, equally stratified by gender (50% male, female), and education level (50% degree or higher, 50% no formal qualifications/don’t know/not applicable, secondary education, high school diploma/A-levels/technical college/community college), which are collected by the platform Prolific upon registration. On Prolific, participants were provided with the study name (“a food ordering study”) and description (“This is a study exploring food choices through delivery apps. You will choose foods and beverages from three different outlets and complete some questionnaires about yourself. Overall the study will take about 10 minutes”), as well as the eligibility criteria.

## Supplementary material ii: Virtual delivery app

For the coffee shop, there were four broad options on the first page: coffee, teas and infusions, hot chocolate and more, and blended ice drinks. Each subsequent page had 8, 4, 3 and 6 drink options respectively. Participants could move backwards and forwards through the pages until they made their choice.


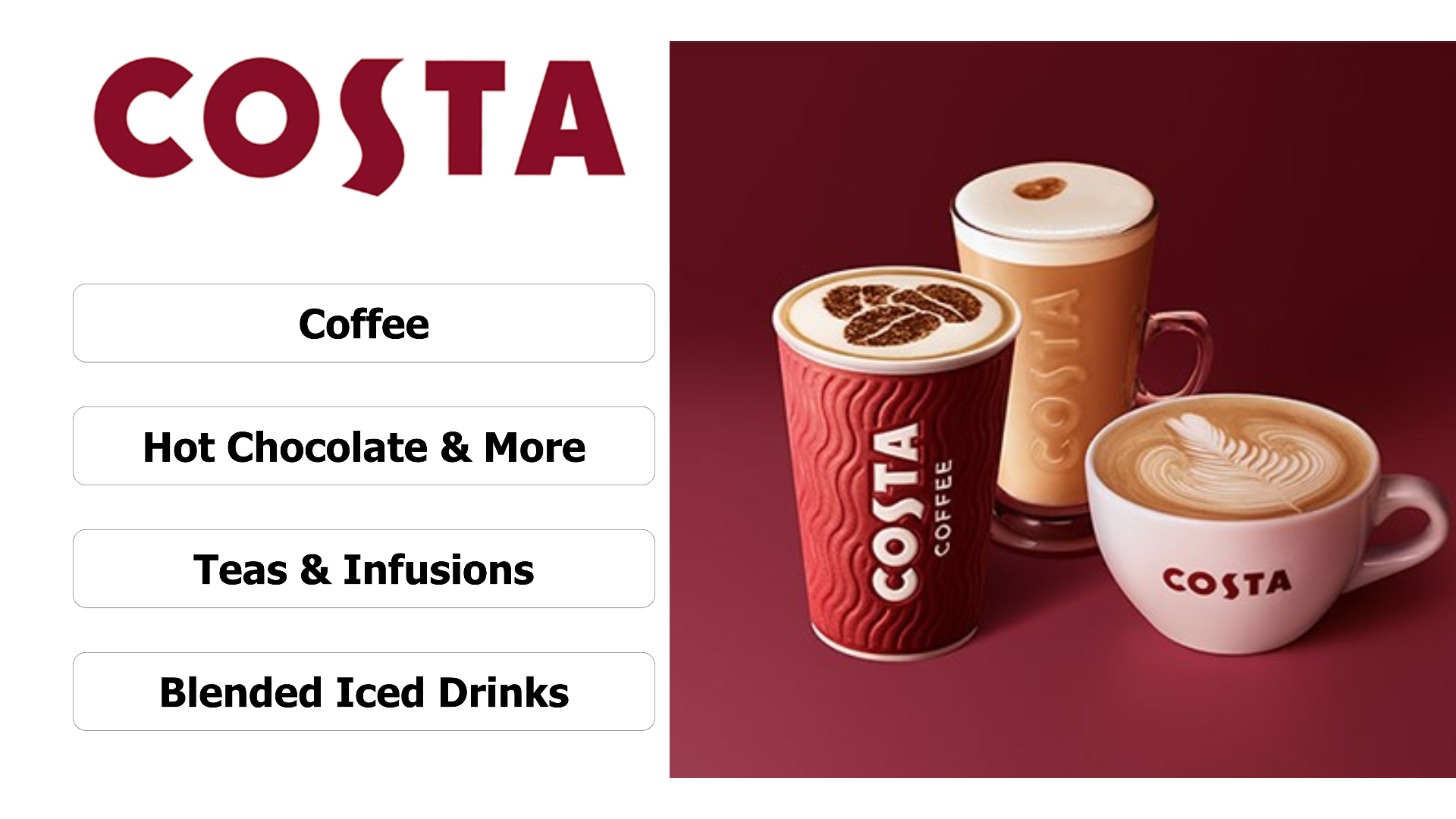

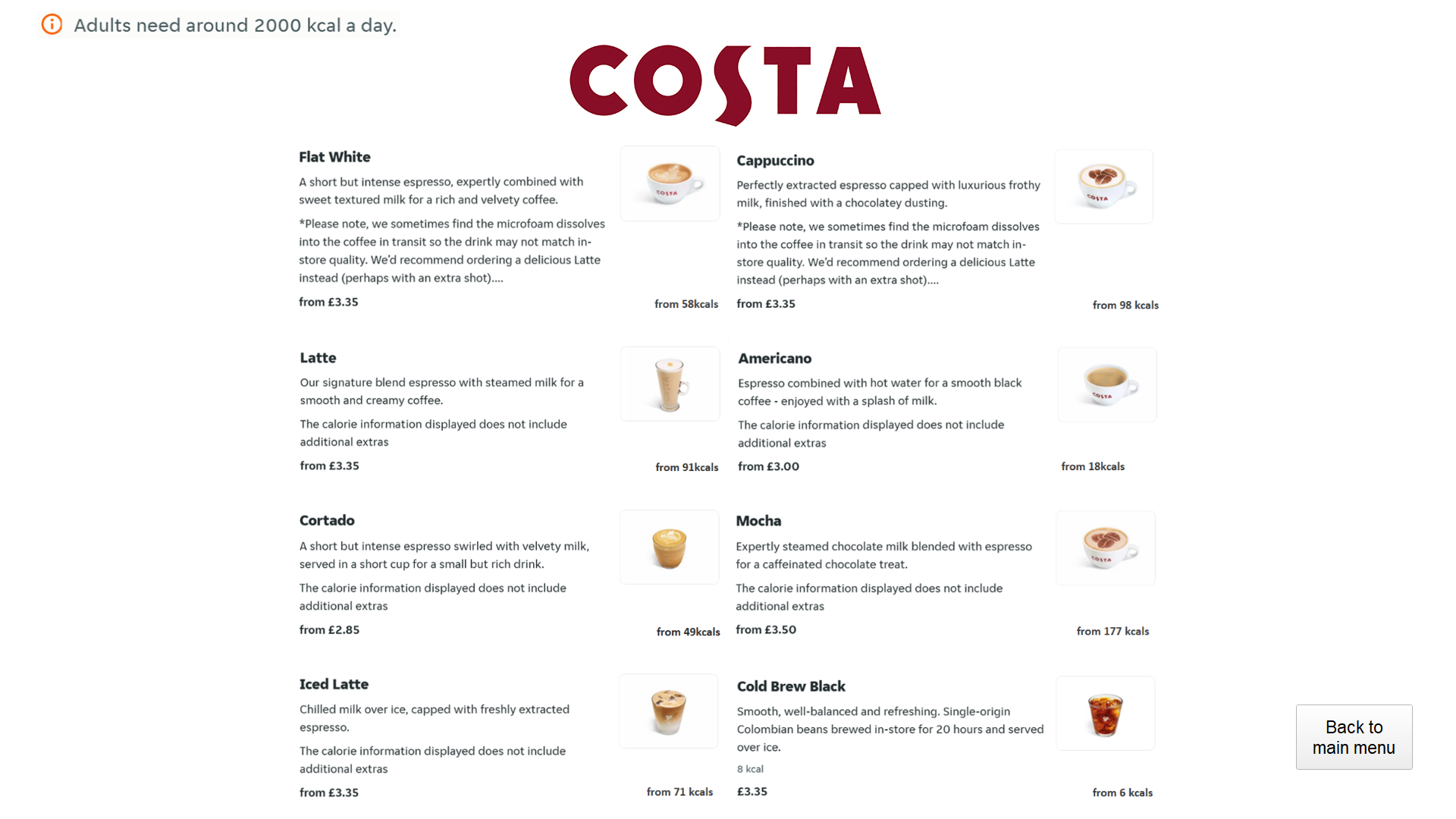


For the sandwich shop, there were 21 possible sandwich options, presented over three pages. Participants could move backwards and forwards through the pages until they made their choice.


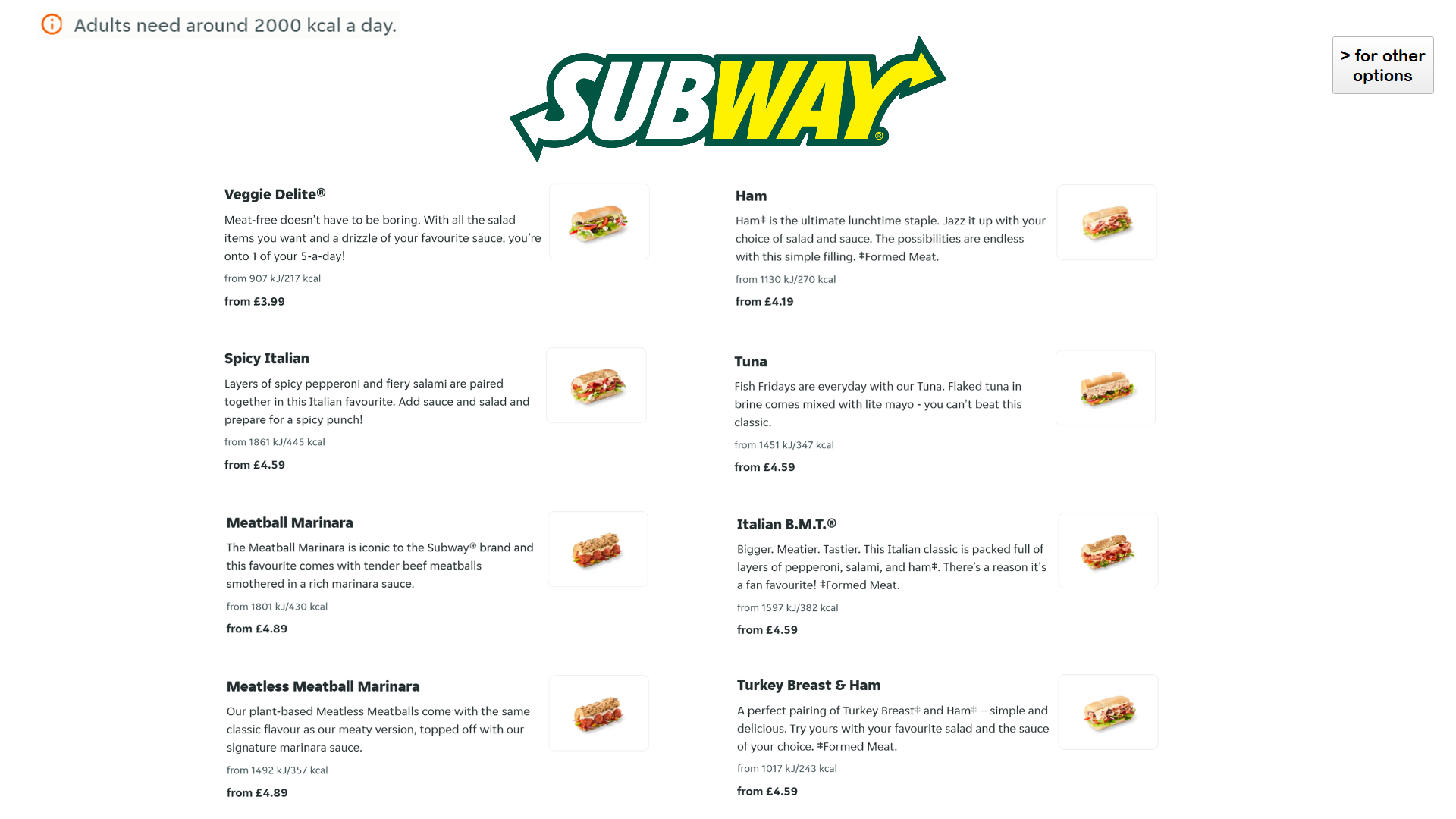


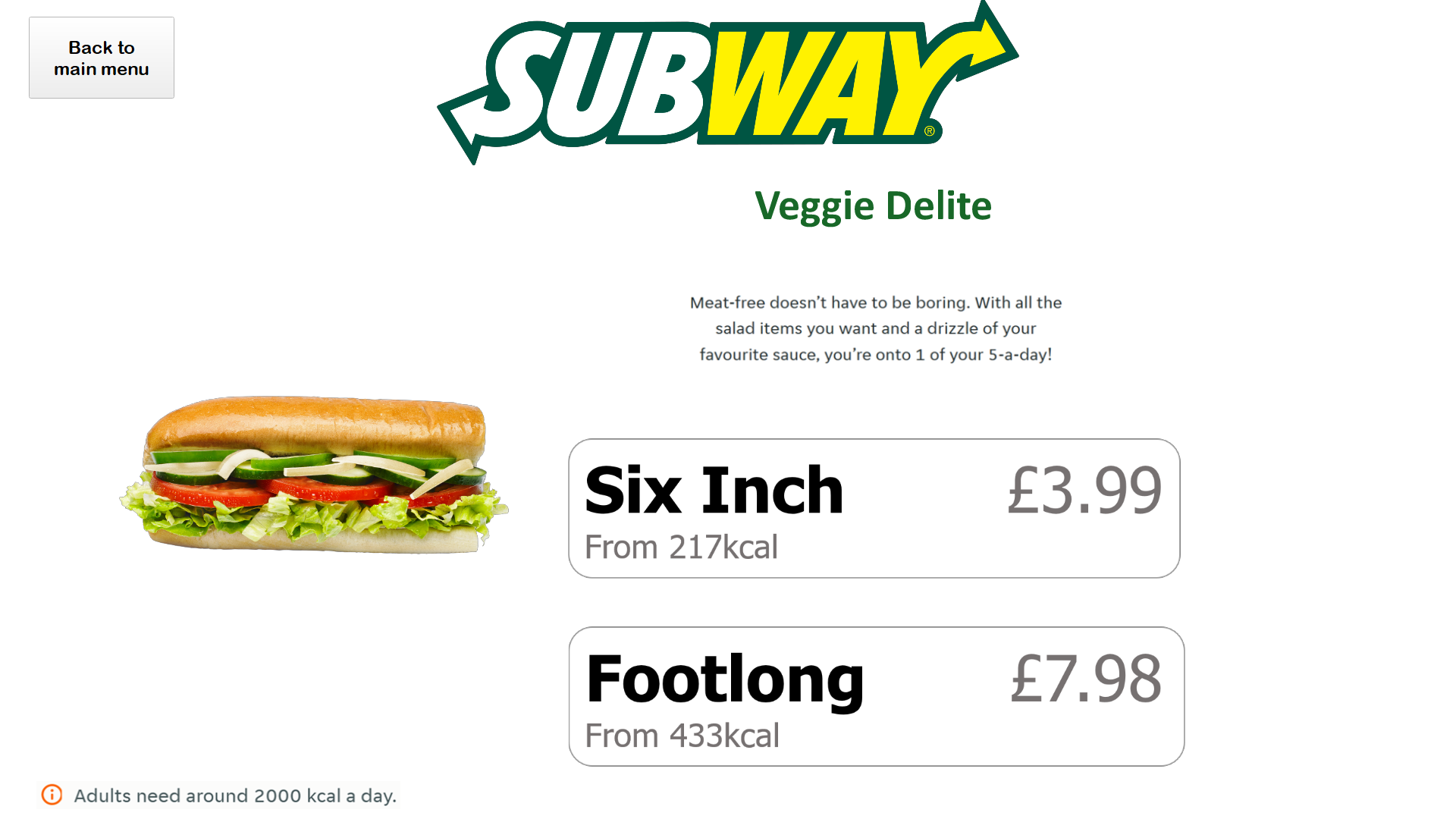


For the fast food outlet, there were 21 possible main meal options (e.g., burgers, wraps), presented over three pages. Participants could move between pages until they made their choice


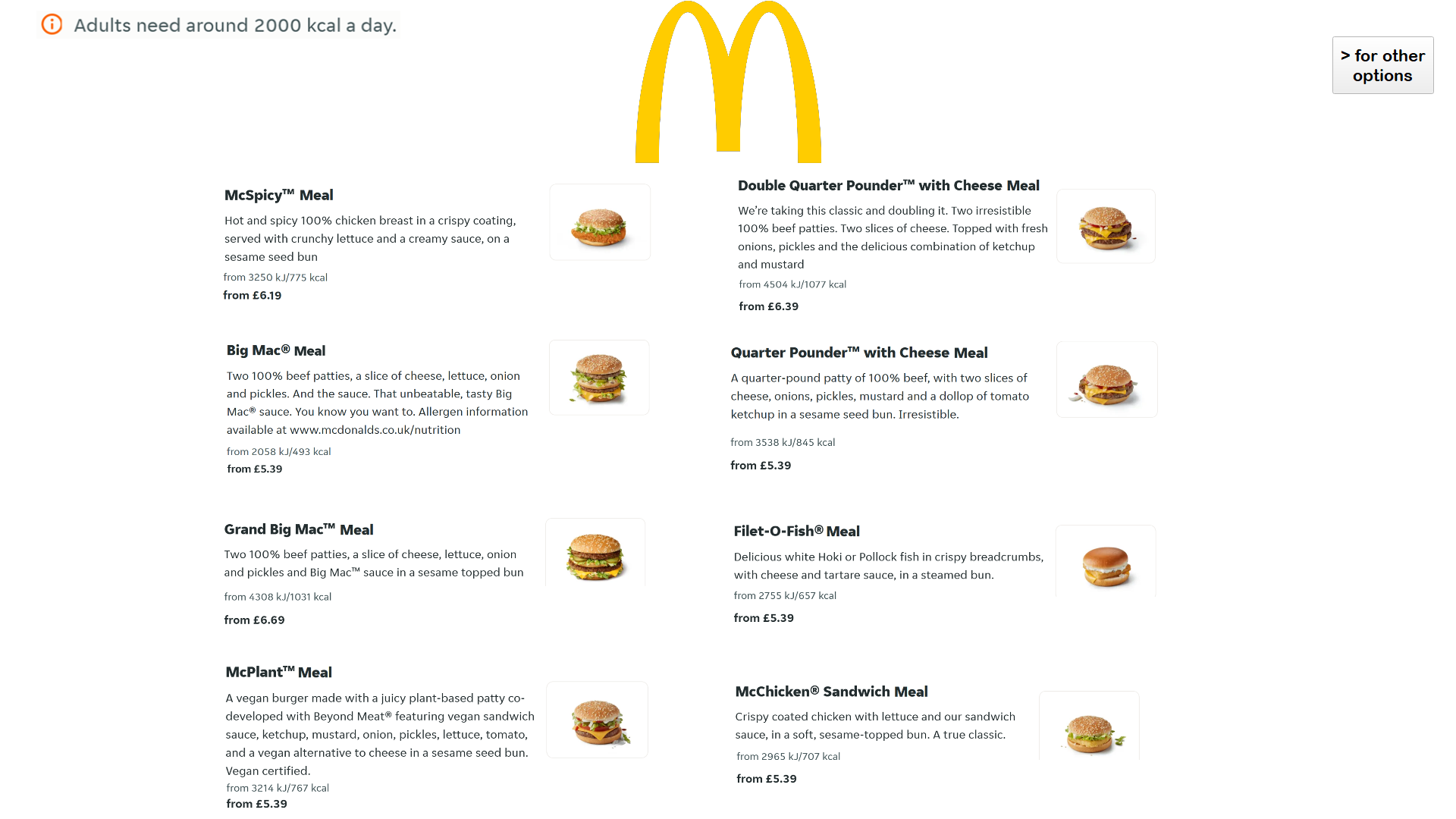


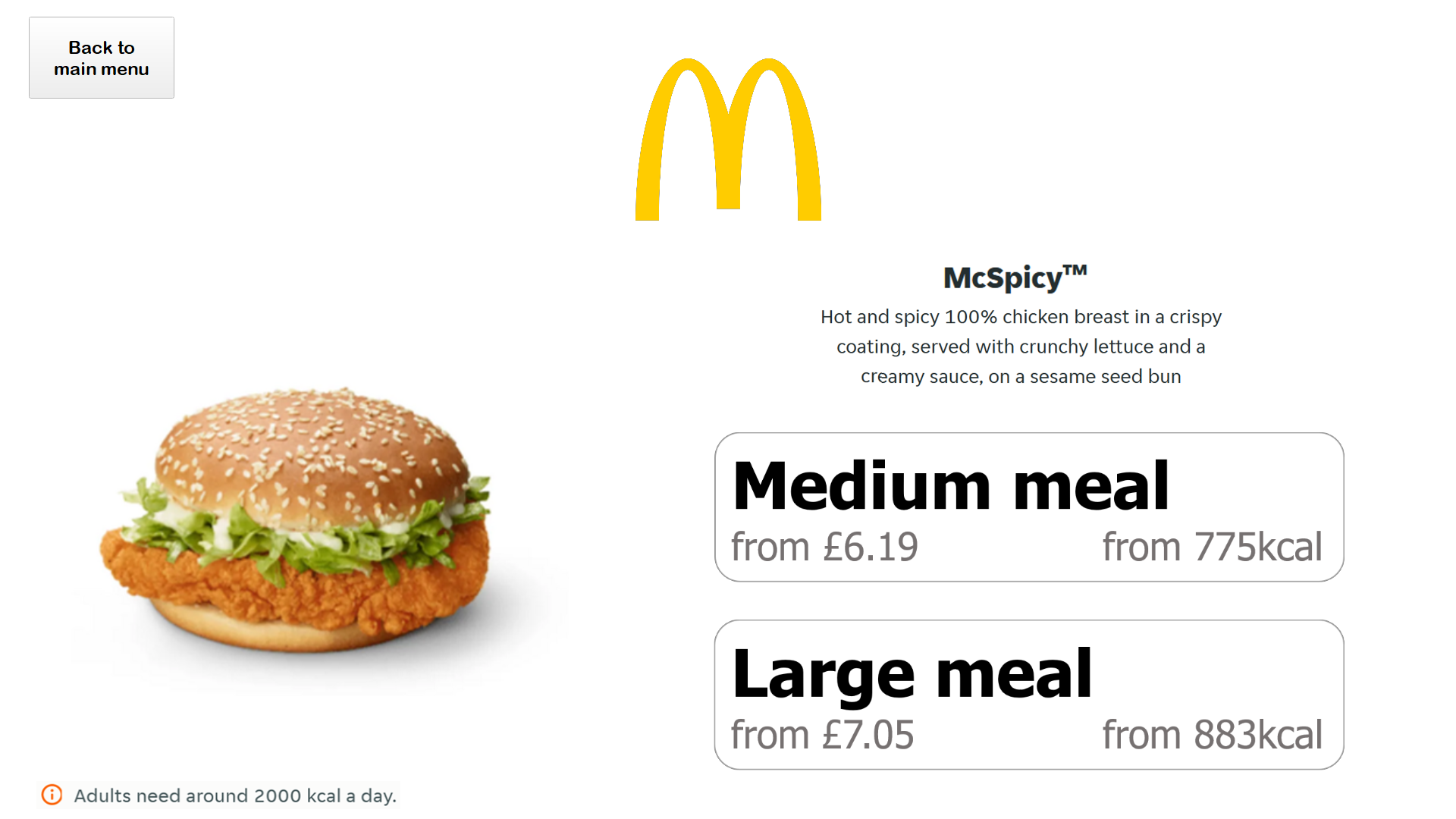


## Supplementary material iii: Example of proportional pricing calculation

*Fast food value pricing*

*Medium meal (775kcal) = £6.19, Large meal (883kcal) = £6.69*

*Proportional pricing.*

*% increase in kcals = 13.94%*

*13.94 % increase in price = £0.86*

*Medium meal = £6.19, Large meal = £7.05*

## Supplementary Material iv: Questionnaire responses

| Question | Strongly disagree | Disagree | Slightly disagree | Neither agree or disagree | Slightly agree | Agree | Strongly Agree |
| --- | --- | --- | --- | --- | --- | --- | --- |
| Do you agree with calorie labelling in the out-of-home food sector? | 64 (5.7%) | 75 (6.7%) | 63 (5.6%) | 133 (11.8%) | 220 (19.5%) | 322 (28.6%) | 249 (22.1%) |
| Do you think that calorie labels would help people to make healthier choices? | 61 (5.4%) | 112 (9.9%) | 92 (8.2%) | 94 (8.3%) | 289 (25.7%) | 294 (26.1%) | 184 (16.3%) |
| Should calorie labelling be introduced in all out-of-home food settings (i.e. including restaurants with <250 employees)? | 90 (8%) | 92 (8.2%) | 89 (7.9%) | 129 (11.5%) | 189 (16.8%) | 295 (26.2%) | 242 (21.5%) |
| Do you agree with the use of proportional pricing in the out-of-home sector? | 78 (6.9%) | 97 (8.6%) | 102 (9.1%) | 166 (14.7%) | 243 (21.6%) | 302 (26.8%) | 138 (12.3%) |
| Do you think proportional pricing would help people to make healthier choices? | 65 (5.8%) | 130 (11.5%) | 99 (8.8%) | 121 (10.7%) | 259 (23%) | 302 (26.8%) | 150 (13.3%) |
|  | | | | | | | |
| I felt that the apps were representative of existing food apps. | 11 (1%) | 22 (2%) | 36 (3.2%) | 76 (6.7%) | 201 (17.9%) | 483 (42.9%) | 297 (26.4%) |
| The food choices I made were influenced by how many calories I thought were in the food options. | 344 (30.6%) | 356 (31.6%) | 131 (11.6%) | 76 (6.8%) | 124 (11%) | 66 (5.9%) | 29 (2.6%) |
| The food choices I made were influenced by the prices of food options. | 195 (17.3%) | 293 (26%) | 162 (14.4%) | 100 (8.9%) | 236 (21%) | 106 (9.4%) | 34 (3.0%) |
| For Costa, my drink choice was typical of what I would normally order. | 20 (1.8%) | 24 (2.1%) | 20 (1.8%) | 49 (4.4%) | 108 (9.6%) | 343 (30.5%) | 562 (49.9%) |
| For Subway, my sandwich choice was typical of what I would normally order. | 15 (1.3%) | 21 (1.9%) | 21 (1.9%) | 57 (5.1%) | 118 (10.5%) | 379 (33.7%) | 515 (45.7%) |
| For McDonalds, my meal choice was typical of what I would normally order | 27 (2.4%) | 23 (2%) | 24 (2.1%) | 35 (3.1%) | 101 (9%) | 354 (31.4%) | 562 (49.9%) |
| **How often do you use the following?** | **Not in the last year** | **Less than once per month** | **1-3 times per month** | **1-2 times per week** | **3 times per week or more** |  |  |
| Food delivery apps | 79 (7%) | 214 (19%) | 614 (55%) | 199 (18%) | 20 (2%) |  |  |
| Costa | 351 (31%) | 462 (41%) | 237 (21%) | 60 (5%) | 16 (1%) |  |  |
| Subway | 485 (43%) | 468 (42%) | 156 (14%) | 17 (2%) | 0 (0%) |  |  |
| McDonalds | 187 (17%) | 389 (35%) | 456 (41%) | 87 (8%) | 7 (<1%) |  |  |

## Supplementary material v: Changes to pre-registered protocol analyses

Minor changes were made to the planned analyses – namely in planned analyses two measures of SEP were mistakenly planned to be included in models. For actual analyses, only educational level was included, and other measures were then explored in the place of education level in sensitivity analyses.

Following peer-review, examination of the main effects of calorie labelling condition, pricing condition and their interaction on combined outlet choices were conducted.

## Supplementary material vi: Sensitivity analyses

| **Outcome** | **Estimate** | **Std. Error** | **Sig.** | **95% CI** | |
| --- | --- | --- | --- | --- | --- |
| **Subjective social status included instead of level of education** | | | | | |
| **Costa size** | | | | | |
| (Intercept) | -0.88 |  |  | -2.13 | 0.37 |
| Labelling condition (kcal labels) | 0.03 | 0.13 | .821 | -0.22 | 0.28 |
| **Pricing condition (proportional pricing)** | **-0.54** | **0.13** | **<.001** | **-0.80** | **-0.29** |
| **Subway size** | | | | | |
| (Intercept) | 0.32 |  |  | -0.99 | 1.64 |
| Labelling condition (kcal labels) | 0.09 | 0.14 | .491 | -0.18 | 0.37 |
| Pricing condition (proportional pricing) | -0.23 | 0.14 | .102 | -0.50 | 0.04 |
| **McDonalds size** | | | | | |
| (Intercept) | 0.42 |  |  | -0.83 | 1.68 |
| Labelling condition (kcal labels) | 0.11 | 0.13 | .382 | -0.14 | 0.36 |
| Pricing condition (proportional pricing) | -0.07 | 0.13 | .590 | -0.32 | 0.18 |
| **Costa kcals** | | | | | |
| (Intercept) | 197.63 |  |  | 125.48 | 269.78 |
| **Labelling condition (kcal labels)** | **-18.92** | **7.17** | **.008** | **-32.98** | **-4.86** |
| Pricing condition (proportional pricing) | -13.68 | 7.25 | .059 | -27.91 | 0.54 |
| **Subway kcals** | | | | | |
| (Intercept) | 869.93 |  |  | 653.56 | 1086.30 |
| Labelling condition (kcal labels) | -0.84 | 20.38 | .967 | -40.83 | 39.15 |
| Pricing condition (proportional pricing) | -1.29 | 20.56 | .950 | -41.64 | 39.06 |
| **McDonalds kcals** | | | | | |
| (Intercept) | 1061.15 |  |  | 903.14 | 1219.16 |
| **Labelling condition (kcal labels)** | **-55.59** | **16.15** | **<.001** | **-87.28** | **-23.90** |
| **Pricing condition (proportional pricing)** | **52.111** | **16.11** | **.001** | **20.51** | **83.72** |
| **Costa spend** | | | | | |
| (Intercept) | 3.80 |  |  | 3.40 | 4.20 |
| Labelling condition (kcal labels) | -0.02 | 0.04 | .579 | -0.10 | 0.06 |
| **Pricing condition (proportional pricing)** | **0.21** | **0.04** | **<.001** | **0.13** | **0.29** |
| **Subway spend** | | | | | |
| (Intercept) | 8.45 |  |  | 6.46 | 10.43 |
| Labelling condition (kcal labels) | -0.02 | 0.19 | .930 | -0.39 | 0.36 |
| **Pricing condition (proportional pricing)** | **0.79** | **0.19** | **<.001** | **0.41** | **1.16** |
| **McDonalds spend** | | | | | |
| (Intercept) | 6.63 |  |  | 5.97 | 7.30 |
| **Labelling condition (kcal labels)** | **-0.17** | **0.07** | **.013** | **-0.31** | **-0.04** |
| **Pricing condition (proportional pricing)** | **0.57** | **0.07** | **<.001** | **0.44** | **0.70** |
| **Equivalised household income included instead of level of education** | | | | | |
| **Costa size** | | | | | |
| (Intercept) | -1.04 |  |  | -2.24 | 0.15 |
| Labelling condition (kcal labels) | 0.02 | 0.13 | .866 | -0.23 | 0.28 |
| **Pricing condition (proportional pricing)** | **-0.54** | **0.13** | **<.001** | **-0.79** | **-0.29** |
| **Subway size** | | | | | |
| (Intercept) | -0.03 |  |  | -1.29 | 1.22 |
| Labelling condition (kcal labels) | 0.09 | 0.14 | .510 | -0.18 | 0.36 |
| Pricing condition (proportional pricing) | -0.23 | 0.14 | .098 | -0.50 | 0.04 |
| **McDonalds size** | | | | | |
| (Intercept) | 0.23 |  |  | -0.96 | 1.42 |
| Labelling condition (kcal labels) | 0.11 | 0.13 | .394 | -0.14 | 0.36 |
| Pricing condition (proportional pricing) | -0.07 | 0.13 | .594 | -0.32 | 0.18 |
| **Costa kcals** | | | | | |
| (Intercept) | 204.99 |  |  | 136.10 | 273.89 |
| **Labelling condition (kcal labels)** | **-17.58** | **7.16** | **.014** | **-31.62** | **-3.54** |
| **Pricing condition (proportional pricing)** | **-14.68** | **7.22** | **.042** | **-28.85** | **-0.50** |
| **Subway kcals** | | | | | |
| (Intercept) | 822.45 |  |  | 608.97 | 1035.93 |
| Labelling condition (kcal labels) | 0.68 | 20.41 | .973 | -39.36 | 40.72 |
| Pricing condition (proportional pricing) | -2.81 | 20.71 | .892 | -43.44 | 37.83 |
| **McDonalds kcals** | | | | | |
| (Intercept) | 1023.80 |  |  | 872.52 | 1175.09 |
| **Labelling condition (kcal labels)** | **-53.12** | **16.17** | **.001** | **-84.85** | **-21.39** |
| **Pricing condition (proportional pricing)** | **49.93** | **16.13** | **.002** | **18.28** | **81.57** |
| **Costa spend** | | | | | |
| (Intercept) | 3.71 |  |  | 3.34 | 4.09 |
| Labelling condition (kcal labels) | -0.02 | 0.04 | .596 | -0.10 | 0.06 |
| **Pricing condition (proportional pricing)** | **0.21** | **0.04** | **<.001** | **0.13** | **0.29** |
| **Subway spend** | | | | | |
| (Intercept) | 7.74 |  |  | 5.75 | 9.74 |
| Labelling condition (kcal labels) | -0.00 | 0.19 | .989 | -0.39 | 0.37 |
| **Pricing condition (proportional pricing)** | **0.77** | **0.19** | **<.001** | **0.39** | **1.15** |
| **McDonalds spend** | | | | | |
| (Intercept) | 6.46 |  |  | 5.82 | 7.10 |
| **Labelling condition (kcal labels)** | **-0.16** | **0.07** | **.018** | **-0.30** | **-0.03** |
| **Pricing condition (proportional pricing)** | **0.56** | **0.07** | **<.001** | **0.43** | **0.70** |
| **Aim guessers removed** | | | | | |
| **Costa size** | | | | | |
| (Intercept) | -1.13 |  |  | -2.36 | 0.10 |
| Labelling condition (kcal labels) | 0.03 | 0.14 | .831 | -0.24 | 0.29 |
| **Pricing condition (proportional pricing)** | **-0.55** | **0.14** | **<.001** | **-0.81** | **-0.28** |
| **Subway size** | | | | | |
| (Intercept) | 0.28 |  |  | -1.00 | 1.57 |
| Labelling condition (kcal labels) | 0.09 | 0.14 | .536 | -0.19 | 0.37 |
| **Pricing condition (proportional pricing)** | **-0.31** | **0.14** | **.034** | **-0.59** | **-0.02** |
| **McDonalds size** | | | | | |
| (Intercept) | 0.28 |  |  | -0.94 | 1.51 |
| Labelling condition (kcal labels) | 0.05 | 0.13 | .710 | -0.21 | 0.31 |
| Pricing condition (proportional pricing) | -0.04 | 0.13 | .746 | -0.31 | 0.22 |
| **Costa kcals** | | | | | |
| (Intercept) | 170.67 |  |  | 99.26 | 242.10 |
| **Labelling condition (kcal labels)** | **-20.00** | **7.56** | **.008** | **-34.82** | **-5.17** |
| Pricing condition (proportional pricing) | -11.45 | 7.58 | .131 | -26.33 | 3.42 |
| **Subway kcals** | | | | | |
| (Intercept) | 841.36 |  |  | 621.71 | 1061.02 |
| Labelling condition (kcal labels) | -1.13 | 21.44 | .958 | -43.21 | 40.95 |
| Pricing condition (proportional pricing) | -7.26 | 21.52 | .736 | -49.49 | 34.98 |
| **McDonalds kcals** | | | | | |
| (Intercept) | 973.86 |  |  | 816.39 | 1131.33 |
| **Labelling condition (kcal labels)** | **-49.36** | **17.00** | **.004** | **-82.72** | **-16.01** |
| **Pricing condition (proportional pricing)** | **53.55** | **16.83** | **.002** | **20.51** | **86.58** |
| **Costa spend** | | | | | |
| (Intercept) | 3.65 |  |  | 3.26 | 4.03 |
| Labelling condition (kcal labels) | -0.02 | 0.04 | .718 | -0.10 | 0.07 |
| **Pricing condition (proportional pricing)** | **0.21** | **0.04** | **<.001** | **0.13** | **0.30** |
| **Subway spend** | | | | | |
| (Intercept) | 7.78 |  |  | 5.71 | 9.84 |
| Labelling condition (kcal labels) | -0.02 | 0.20 | .931 | -0.41 | 0.37 |
| **Pricing condition (proportional pricing)** | **0.72** | **0.20** | **<.001** | **0.33** | **1.11** |
| **McDonalds spend** | | | | | |
| (Intercept) | 6.24 |  |  | 5.58 | 6.90 |
| **Labelling condition (kcal labels)** | **-0.16** | **0.07** | **.025** | **-0.31** | **-0.02** |
| **Pricing condition (proportional pricing)** | **0.60** | **0.07** | **<.001** | **0.46** | **0.74** |
| **Missing BMI removed** | | | | | |
| **Costa size** | | | | | |
| (Intercept) | -0.97 |  |  | 2.15 | 0.20 |
| Labelling condition (kcal labels) | 0.02 | 0.13 | .853 | -0.23 | 0.28 |
| **Pricing condition (proportional pricing)** | **-0.54** | **0.13** | **<.001** | **-0.80** | **-0.29** |
| **Subway size** | | | | | |
| (Intercept) | -0.01 |  |  | -1.24 | 1.23 |
| Labelling condition (kcal labels) | 0.09 | 0.14 | .524 | -0.18 | 0.36 |
| Pricing condition (proportional pricing) | -0.23 | 0.14 | .091 | -0.50 | 0.04 |
| **McDonalds size** | | | | | |
| (Intercept) | 0.30 |  |  | -0.87 | 1.48 |
| Labelling condition (kcal labels) | 0.11 | 0.13 | .373 | -0.14 | 0.37 |
| Pricing condition (proportional pricing) | -0.07 | 0.13 | .589 | -0.32 | 0.18 |
| **Costa kcals** | | | | | |
| (Intercept) | 177.91 |  |  | 109.49 | 246.33 |
| **Labelling condition (kcal labels)** | **-19.15** | **7.18** | **.008** | **-33.24** | **-5.06** |
| Pricing condition (proportional pricing) | -14.01 | 7.24 | .053 | -28.21 | 0.18 |
| **Subway kcals** | | | | | |
| (Intercept) | 802.61 |  |  | 595.60 | 1009.63 |
| Labelling condition (kcal labels) | -0.76 | 20.36 | .970 | -40.71 | 39.19 |
| Pricing condition (proportional pricing) | -2.21 | 20.57 | .915 | -42.58 | 38.16 |
| **McDonalds kcals** | | | | | |
| (Intercept) | 1002.62 |  |  | 852.64 | 1152.59 |
| **Labelling condition (kcal labels)** | **-53.76** | **16.21** | **<.001** | **-85.56** | **-21.97** |
| **Pricing condition (proportional pricing)** | **51.80** | **16.12** | **.001** | **20.17** | **83.44** |
| **Costa spend** | | | | | |
| (Intercept) | 3.70 |  |  | 3.33 | 4.06 |
| Labelling condition (kcal labels) | -0.02 | 0.04 | .547 | -0.11 | 0.06 |
| **Pricing condition (proportional pricing)** | **0.20** | **0.04** | **<.001** | **0.12** | **0.28** |
| **Subway spend** | | | | | |
| (Intercept) | 7.43 |  |  | 5.48 | 9.39 |
| Labelling condition (kcal labels) | -0.02 | 0.19 | .926 | -0.39 | 0.36 |
| **Pricing condition (proportional pricing)** | **0.78** | **0.19** | **<.001** | **0.41** | **1.16** |
| **McDonalds spend** | | | | | |
| (Intercept) | 6.38 |  |  | 5.76 | 7.01 |
| **Labelling condition (kcal labels)** | **-0.17** | **0.07** | **.016** | **-0.30** | **-0.03** |
| **Pricing condition (proportional pricing)** | **0.57** | **0.07** | **<.001** | **0.43** | **0.70** |

Secondary analyses were not conducted for gender (other) due to the small sample size (N=6).

## Supplementary material vii: Sample size calculation

A previous study assessing the impact of pricing strategy on calorie purchase (1) found that being presented with standard pricing (compared with any-size-same pricing) led to a mean reduction in calories ordered of 26.76kcals from non-diet beverages and the effect size was equivalent to f = 0.24 (medium sized statistical effect). However, the previous study examined beverages, as opposed to the product ranges being examined in the present study and therefore the estimated effect size of proportional pricing may be smaller in the present study.

We powered the present study to detect a medium effect size of f=0.24 through regression analyses with four groups and 10 covariates at 0.80% power and an error probability of 0.05. This required a sample size of n=292. Assuming that the effect of proportional pricing may be statistically small and that sample sizes to detect an interaction in regression analysis (e.g. interaction between proportional pricing and calorie labelling) is advised to be x4 times larger than that of a main effect (2), in the present study we aimed to recruit N=1200 participants.

Across a series of power analyses this sample size provides sufficient power to detect small effects of proportional pricing, calorie labelling, their interaction and participant characteristic interactions with proportional pricing using both logistic and linear regression (as planned). Furthermore, the sample size will provide a suitably large number of participants to provide a reliable estimate of support for policies (sample sizes of 1000 or more are considered appropriate to minimize sampling error for representative samples). An N=1200 participants accounts for loss of up to approximately 15% whilst allowing for reasonable numbers of participants per strata in stratified recruitment (i.e. 50% male/female, 50% higher vs. lower SES).

## Supplementary Material viii: Participant food and beverage orders overall and by condition: Size choice, kcals ordered and hypothetical spend

|  | **Overall** | **Condition 1**  **No kcal labels**  **Value price** | **Condition 2**  **No kcal labels**  **Proportional** | **Condition 3**  **Kcal labels**  **Value price** | **Condition 4**  **Kcal labels**  **Proportional** |
| --- | --- | --- | --- | --- | --- |
| **Coffee shop**  **size** | Medium=717 (64%)  Large=409 (36%) | Medium=162 (58%)  Large=115 (42%) | Medium=203 (69%)  Large=91 (31%) | Medium=150 (56%)  Large=120 (44%) | Medium=202 (71%)  Large=83 (29%) |
| **Sandwich shop size** | 6 inch=740 (66%)  12 inch=386 (34%) | 6 inch=174 (63%)  12 inch=103 (37%) | 6 inch=202 (69%)  12 inch=92 (31%) | 6 inch=169 (63%)  12 inch=101 (37%) | 6 inch=195 (68%)  12 inch=90 (31%) |
| **Fast food size** | Medium=571 (51%)  Large=555 (49%) | Medium=139 (50%)  Large=138 (50%) | Medium=155 (53%)  Large=139 (47%) | Medium=130 (48%)  Large=140 (52%) | Medium=147 (52%)  Large=138 (48%) |
|  | | | | | |
| **Coffee shop**  **kcals** | 173.51 (125.68) | 182.78  (131.20) | 182.19 (125.33) | 179.09 (125.13) | 150.25 (118.63) |
| **Sandwich shop kcals** | 764.04 (362.87) | 784.48 (365.33) | 754.65 (336.79) | 759.33 (365.53) | 758.30 (384.40) |
| **Fast food kcals** | 1037.56 (296.53) | 1032.88 (275.34) | 1100.79 (316.03) | 999.63 (268.94) | 1012.81 (311.35) |
| **Coffee shop spend** | 3.70 (0.72) | 3.57 (0.54) | 3.85 (0.82) | 3.62 (0.56) | 3.74 (0.84) |
| **Sandwich shop spend** | 8.71 (3.37) | 8.50 (2.96) | 8.98 (3.57) | 8.19 (2.87) | 9.11 (3.88) |
| **Fast food spend** | 6.58 (1.23) | 6.37 (0.97) | 6.96 (1.37) | 6.25 (0.96) | 6.70 (1.40) |

Size choices are presented as the number of participants making each choice and percentages. Kcals ordered and hypothetical spend are presented as means and standard deviations.

## Supplementary Material ix: Secondary analyses

| **Outcome** | **Estimate** | **Std. Error** | **Sig.** | **99% CI** | |
| --- | --- | --- | --- | --- | --- |
|  |  |  |  | **Lower** | **Upper** |
| **Coffee shop size choice** | | | | | |
| (Intercept) | -0.50 |  |  | -0.94 | 1.94 |
| Kcal labels*BMI | -0.00 | 0.02 | .985 | -0.05 | 0.05 |
| Proportional pricing*BMI | 0.01 | 0.02 | .485 | -0.04 | 0.07 |
| (Intercept) | -1.54 |  |  | -3.10 | 0.00 |
| Kcal labels*Age | -0.00 | 0.01 | .863 | -0.03 | 0.02 |
| Proportional pricing*Age | 0.00 | 0.01 | .924 | -0.02 | 0.03 |
| (Intercept) | -1.61 |  |  | -3.31 | 0.07 |
| Kcal labels*Health | -0.02 | 0.21 | .942 | -0.55 | 0.52 |
| Proportional pricing*Health | 0.23 | 0.21 | .258 | -0.30 | 0.78 |
| (Intercept) | 0.07 |  |  | -1.45 | 1.59 |
| Kcal labels*Sensory | -0.28 | 0.25 | .270 | -0.92 | 0.37 |
| Proportional pricing*Sensory | -0.12 | 0.25 | .632 | -0.53 | 0.77 |
| (Intercept) | -1.46 |  |  | -2.91 | -0.03 |
| Kcal labels*Price | -0.12 | 0.22 | .599 | -0.68 | 0.45 |
| Proportional pricing*Price | -0.20 | 0.22 | .376 | -0.77 | 0.37 |
| (Intercept) | -1.01 |  |  | -2.65 | 0.61 |
| Kcal labels*Weight | 0.27 | 0.18 | .144 | -0.20 | 0.74 |
| Proportional pricing*Weight | -0.02 | 0.18 | .917 | -0.49 | 0.45 |
| (Intercept) | -1.01 |  |  | -2.61 | 0.58 |
| Kcal labels*Familiarity | -0.06 | 0.18 | .725 | -0.53 | 0.40 |
| Proportional pricing*Familiarity | 0.17 | 0.18 | .357 | -0.30 | 0.64 |
| (Intercept) | -0.86 |  |  | -2.43 | 0.70 |
| Kcal labels*Education (university) | 0.26 | 0.26 | .320 | -0.41 | 0.93 |
| Proportional pricing*Education (university) | 0.25 | 0.26 | .338 | -0.42 | 0.92 |
| (Intercept) | -0.96 |  |  | -2.52 | 0.59 |
| Kcal labels*Ethnicity (non-white) | 0.14 | 0.37 | .710 | -0.82 | 1.10 |
| Proportional pricing*Ethnicity (non-white) | 0.12 | 0.37 | .757 | -0.86 | 1.07 |
| (Intercept) | -1.09 |  |  | -2.66 | 0.46 |
| Kcal labels*Gender (Woman) | -0.28 | 0.26 | .288 | -0.94 | 0.39 |
| Proportional pricing*Gender (Woman) | -0.31 | 0.26 | .240 | -0.98 | 0.36 |
| **Sandwich shop size choice** | | | | | |
| Intercept | 1.59 |  |  | -0.08 | 3.12 |
| Kcal labels*BMI | -0.06 | 0.02 | .782 | -0.06 | 0.05 |
| Proportional pricing*BMI | 0.04 | 0.02 | .091 | -0.02 | 0.09 |
| (Intercept) | -2.13 |  |  | -3.78 | -0.49 |
| Kcal labels*Age | 0.02 | 0.01 | .035 | -0.01 | 0.05 |
| Proportional pricing*Age | 0.00 | 0.01 | .658 | -0.02 | 0.03 |
| (Intercept) | -0.01 |  |  | -1.79 | 1.76 |
| Kcal labels*Health | 0.24 | 0.22 | .287 | -0.34 | 0.82 |
| Proportional pricing*Health | 0.52 | 0.22 | .021 | -0.06 | 1.10 |
| (Intercept) | 0.64 |  |  | -0.99 | 2.27 |
| Kcal labels*Sensory | -0.03 | 0.26 | .923 | -0.70 | 0.65 |
| Proportional pricing*Sensory | 0.56 | 0.26 | .033 | -0.12 | 1.25 |
| (Intercept) | -0.26 |  |  | -1.78 | 1.24 |
| Kcal labels*Price | -0.00 | 0.24 | .995 | -0.61 | 0.60 |
| Proportional pricing*Price | 0.14 | 0.24 | .559 | -0.47 | 0.75 |
| (Intercept) | -0.37 |  |  | -2.09 | 1.34 |
| Kcal labels*Weight | -0.13 | 0.19 | .493 | -0.64 | 0.37 |
| Proportional pricing*Weight | 0.12 | 0.20 | .542 | -0.38 | 0.62 |
| (Intercept) | 0.26 |  |  | -1.42 | 1.94 |
| Kcal labels*Familiarity | 0.06 | 0.19 | .740 | -0.43 | 0.56 |
| Proportional pricing*Familiarity | 0.01 | 0.20 | .958 | -0.50 | 0.52 |
| (Intercept) | -0.06 |  |  | -1.70 | 1.58 |
| Kcal labels*Education (university) | -0.12 | 0.28 | .677 | -0.83 | 0.60 |
| Proportional pricing*Education (university) | -0.06 | 0.28 | .820 | -0.78 | 0.65 |
| (Intercept) | 0.02 |  |  | -1.61 | 1.65 |
| Kcal labels*Ethnicity (non-white) | 0.28 | 0.39 | .472 | -0.73 | 1.30 |
| Proportional pricing*Ethnicity (non-white) | 0.33 | 0.39 | .405 | -0.69 | 1.34 |
| (Intercept) | -0.00 |  |  | -1.64 | 1.64 |
| Kcal labels*Gender (Woman) | -0.18 | 0.28 | .522 | -0.90 | 0.54 |
| Proportional pricing*Gender (Woman) | 0.23 | 0.28 | .414 | -0.49 | 0.96 |
| **Fast food size choice** | | | | | |
| (Intercept) | 1.71 |  |  | 0.28 | 3.15 |
| Kcal labels*BMI | 0.02 | 0.02 | .460 | -0.04 | 0.07 |
| Proportional pricing*BMI | 0.02 | 0.02 | .327 | -0.03 | 0.08 |
| (Intercept) | -0.98 |  |  | -2.53 | 0.57 |
| Kcal labels*Age | 0.01 | 0.01 | .580 | -0.02 | 0.03 |
| Proportional pricing*Age | -0.00 | 0.01 | .811 | -0.03 | 0.02 |
| (Intercept) | 0.30 |  |  | -1.37 | 1.97 |
| Kcal labels*Health | -0.19 | 0.21 | .370 | -0.72 | 0.35 |
| Proportional pricing*Health | -0.01 | 0.21 | .978 | -0.54 | 0.53 |
| (Intercept) | 0.82 |  |  | -0.80 | 2.35 |
| Kcal labels*Sensory | 0.03 | 0.25 | .900 | -0.60 | 0.66 |
| Proportional pricing*Sensory | 0.09 | 0.25 | .718 | -0.55 | 0.73 |
| (Intercept) | 0.23 |  |  | -1.20 | 1.66 |
| Kcal labels*Price | 0.18 | 0.22 | .422 | -0.39 | 0.74 |
| Proportional pricing*Price | -0.08 | 0.22 | .720 | -0.65 | 0.49 |
| (Intercept) | -0.14 |  |  | -1.77 | 1.49 |
| Kcal labels*Weight | -0.16 | 0.18 | .382 | -0.63 | 0.31 |
| Proportional pricing*Weight | -0.25 | 0.18 | .165 | -0.72 | 0.22 |
| (Intercept) | 0.58 |  |  | -1.01 | 2.17 |
| Kcal labels*Familiarity | 0.08 | 0.18 | .645 | -0.38 | 0.55 |
| Proportional pricing*Familiarity | -0.04 | 0.18 | .821 | -0.52 | 0.43 |
| (Intercept) | 0.27 |  |  | -1.29 | 1.83 |
| Kcal labels*Education (university) | -0.18 | 0.26 | .494 | -0.84 | 0.49 |
| Proportional pricing*Education (university) | 0.02 | 0.26 | .946 | -0.65 | 0.68 |
| (Intercept) | 0.33 |  |  | -1.21 | 1.89 |
| Kcal labels*Ethnicity (non-white) | 0.24 | 0.37 | .514 | -0.72 | 1.21 |
| Proportional pricing*Ethnicity (non-white) | -0.09 | 0.37 | .812 | -1.05 | 0.87 |
| (Intercept) | 0.17 |  |  | -1.39 | 1.73 |
| Kcal labels*Gender (Woman) | -0.64 | 0.26 | .013 | -1.31 | 0.02 |
| Proportional pricing*Gender (Woman) | 0.08 | 0.26 | .764 | -0.59 | 0.74 |
| **Coffee shop kcals ordered** | | | | | |
| (Intercept) | 231.28 |  |  | 150.28 | 312.29 |
| Kcal labels*BMI | -1.94 | 1.18 | .098 | -4.98 | 1.09 |
| Proportional pricing*BMI | 0.87 | 1.17 | .458 | -2.16 | 3.90 |
| (Intercept) | 133.99 |  |  | 43.68 | 224.30 |
| Kcal labels*Age | -0.01 | 0.52 | .990 | -1.34 | 1.33 |
| Proportional pricing*Age | -0.75 | 0.52 | .147 | -2.09 | 0.59 |
| (Intercept) | 88.43 |  |  | -6.15 | 183.00 |
| Kcal labels*Health | 25.03 | 11.99 | .037 | -5.91 | 55.96 |
| Proportional pricing*Health | 16.66 | 11.92 | .163 | -14.11 | 47.42 |
| (Intercept) | 213.51 |  |  | 127.20 | 299.82 |
| Kcal labels*Sensory | -3.71 | 14.22 | .794 | -40.41 | 32.99 |
| Proportional pricing*Sensory | -13.18 | 14.33 | .358 | -50.16 | 23.80 |
| (Intercept) | 161.03 |  |  | 75.87 | 246.19 |
| Kcal labels*Price | -6.06 | 12.44 | .626 | -38.15 | 26.03 |
| Proportional pricing*Price | -3.54 | 12.41 | .775 | -35.55 | 28.47 |
| (Intercept) | 175.43 |  |  | 80.31 | 270.54 |
| **Kcal labels*Weight** | **29.57** | **9.70** | **.002** | **4.53** | **54.60** |
| Proportional pricing*Weight | -8.55 | 9.77 | .382 | -33.76 | 16.66 |
| (Intercept) | 241.91 |  |  | 152.37 | 331.45 |
| Kcal labels*Familiarity | -13.78 | 10.38 | .184 | -40.56 | 12.99 |
| Proportional pricing*Familiarity | 3.95 | 10.39 | .704 | -22.87 | 30.77 |
| (Intercept) | 177.28 |  |  | 85.61 | 268.95 |
| Kcal labels*Education (university) | 8.88 | 14.42 | .538 | -28.33 | 46.08 |
| Proportional pricing*Education (university) | -6.90 | 14.49 | .634 | -44.28 | 30.47 |
| (Intercept) | 179.22 |  |  | 88.85 | 269.59 |
| Kcal labels*Ethnicity (non-white) | 5.93 | 23.19 | .798 | -53.90 | 65.76 |
| Proportional pricing*Ethnicity (non-white) | 52.44 | 23.00 | .023 | -6.90 | 111.79 |
| (Intercept) | 178.23 |  |  | 87.46 | 269.00 |
| Kcal labels*Gender (Woman) | 11.24 | 14.35 | .434 | -25.78 | 48.25 |
| Proportional pricing*Gender (Woman) | -10.91 | 14.39 | .448 | -48.04 | 26.22 |
| **Sandwich shop kcals ordered** | | | | | |
| (Intercept) | 1080.61 |  |  | 840.19 | 1321.03 |
| Kcal labels*BMI | 0.41 | 3.78 | .913 | -9.34 | 10.16 |
| Proportional pricing*BMI | 5.66 | 3.79 | .135 | -4.11 | 15.43 |
| (Intercept) | 516.63 |  |  | 244.06 | 789.21 |
| Kcal labels*Age | -0.49 | 1.48 | .739 | -4.31 | 3.32 |
| Proportional pricing*Age | 1.07 | 1.48 | .470 | -2.75 | 4.90 |
| (Intercept) | 910.98 |  |  | 647.85 | 1174.12 |
| Kcal labels*Health | -10.01 | 33.30 | .764 | -95.92 | 75.91 |
| Proportional pricing*Health | 43.95 | 33.84 | .194 | -43.36 | 131.26 |
| (Intercept) | 932.69 |  |  | 667.59 | 1197.80 |
| Kcal labels*Sensory | 10.85 | 39.90 | .786 | -92.11 | 113.81 |
| Proportional pricing*Sensory | 28.15 | 40.13 | .483 | -75.40 | 131.71 |
| (Intercept) | 772.79 |  |  | 523.67 | 1021.91 |
| Kcal labels*Price | 48.16 | 36.06 | .182 | -44.89 | 141.21 |
| Proportional pricing*Price | -8.48 | 36.13 | .815 | -101.71 | 84.75 |
| (Intercept) | 624.19 |  |  | 316.50 | 931.87 |
| Kcal labels*Weight | -13.83 | 27.82 | .619 | -85.60 | 57.94 |
| Proportional pricing*Weight | 3.07 | 28.41 | .914 | -70.24 | 76.38 |
| (Intercept) | 791.16 |  |  | 527.62 | 1054.70 |
| Kcal labels*Familiarity | 53.25 | 30.20 | .078 | -24.68 | 131.18 |
| Proportional pricing*Familiarity | -13.50 | 30.03 | .653 | -90.98 | 63.98 |
| (Intercept) | 813.01 |  |  | 538.46 | 1087.56 |
| Kcal labels*Education (university) | 14.25 | 40.93 | .728 | -91.37 | 119.87 |
| Proportional pricing*Education (university) | 33.78 | 41.23 | .413 | -72.60 | 140.15 |
| (Intercept) | 805.27 |  |  | 530.92 | 1079.63 |
| Kcal labels*Ethnicity (non-white) | 28.96 | 72.28 | .689 | -157.54 | 215.45 |
| Proportional pricing*Ethnicity (non-white) | 15.34 | 72.14 | .832 | -170.81 | 201.49 |
| (Intercept) | 799.29 |  |  | 523.92 | 1074.65 |
| Kcal labels*Gender (Woman) | 0.48 | 40.82 | .991 | -104.85 | 105.81 |
| Proportional pricing*Gender (Woman) | -15.75 | 41.37 | .704 | -122.49 | 90.99 |
| **Fast food kcals ordered** | | | | | |
| (Intercept) | 1253.61 |  |  | 1118.14 | 1389.08 |
| Kcal labels*BMI | -6.93 | 2.90 | .017 | -12.62 | -1.24 |
| Proportional pricing*BMI | 0.43 | 2.97 | .885 | -5.40 | 6.27 |
| (Intercept) | 840.72 |  |  | 647.15 | 1034.29 |
| Kcal labels*Age | -1.72 | 1.19 | .149 | -4.77 | 1.35 |
| Proportional pricing*Age | -1.30 | 1.18 | .271 | -4.33 | 1.74 |
| (Intercept) | 952.94 |  |  | 747.42 | 1158.46 |
| Kcal labels*Health | 13.29 | 27.38 | .627 | -57.35 | 83.93 |
| Proportional pricing*Health | -25.77 | 27.42 | .348 | -96.53 | 44.99 |
| (Intercept) | 1231.26 |  |  | 1029.01 | 1433.51 |
| Kcal labels*Sensory | -11.82 | 31.63 | .709 | -93.43 | 69.80 |
| Proportional pricing*Sensory | -34.99 | 31.63 | .269 | -116.60 | 46.62 |
| (Intercept) | 1032.20 |  |  | 835.80 | 1128.60 |
| Kcal labels*Price | 27.35 | 27.05 | .312 | -42.44 | 97.14 |
| Proportional pricing*Price | 6.06 | 27.06 | .823 | -63.75 | 75.87 |
| (Intercept) | 819.95 |  |  | 611.40 | 1028.50 |
| Kcal labels*Weight | 1.91 | 23.05 | .934 | -57.56 | 61.38 |
| Proportional pricing*Weight | -45.55 | 22.89 | .047 | -104.60 | 13.50 |
| (Intercept) | 1037.85 |  |  | 840.14 | 1235.56 |
| Kcal labels*Familiarity | -27.79 | 24.02 | .248 | -89.77 | 34.20 |
| Proportional pricing*Familiarity | -15.73 | 24.41 | .519 | -78.71 | 47.24 |
| (Intercept) | 1007.36 |  |  | 805.66 | 1209.05 |
| Kcal labels*Education (university) | -6.66 | 32.19 | .836 | -89.70 | 76.39 |
| Proportional pricing*Education (university) | 16.95 | 32.32 | .600 | -66.43 | 100.34 |
| (Intercept) | 1014.72 |  |  | 816.06 | 1213.37 |
| Kcal labels*Ethnicity (non-white) | 65.77 | 46.84 | .161 | -55.08 | 186.62 |
| Proportional pricing*Ethnicity (non-white) | 60.51 | 47.36 | .202 | -61.69 | 182.70 |
| (Intercept) | 1006.83 |  |  | 808.56 | 1205.09 |
| Kcal labels*Gender (Woman) | 6.76 | 32.22 | .834 | -76.38 | 89.89 |
| Proportional pricing*Gender (Woman) | 11.86 | 32.20 | .713 | -71.22 | 94.93 |
| **Coffee shop spend** | | | | | |
| (Intercept) | 4.07 |  |  | 3.64 | 4.50 |
| Kcal labels*BMI | -0.01 | 0.01 | .372 | -0.02 | 0.01 |
| **Proportional pricing*BMI** | **0.02** | **0.01** | **.003** | **0.00** | **0.04** |
| (Intercept) | 3.22 |  |  | 2.75 | 3.69 |
| Kcal labels*Age | -0.00 | 0.00 | .822 | -0.01 | 0.01 |
| Proportional pricing*Age | -0.00 | 0.00 | .104 | -0.01 | 0.00 |
| (Intercept) | 3.52 |  |  | 3.00 | 4.04 |
| Kcal labels*Health | -0.04 | 0.07 | .494 | -0.21 | 0.12 |
| Proportional pricing*Health | -0.00 | 0.07 | .958 | -0.17 | 0.17 |
| (Intercept) | 3.94 |  |  | 3.45 | 4.44 |
| Kcal labels*Sensory | -0.19 | 0.08 | .014 | -0.40 | 0.01 |
| Proportional pricing*Sensory | -0.02 | 0.08 | .781 | -0.22 | 0.18 |
| (Intercept) | 3.44 |  |  | 2.99 | 3.88 |
| Kcal labels*Price | -0.07 | 0.07 | .308 | -0.26 | 0.11 |
| Proportional pricing*Price | -0.06 | 0.07 | .371 | -0.24 | 0.12 |
| (Intercept) | 3.72 |  |  | 3.19 | 4.25 |
| Kcal labels*Weight | 0.02 | 0.06 | .671 | -0.12 | 0.17 |
| Proportional pricing*Weight | -0.01 | 0.06 | .849 | -0.15 | 0.13 |
| (Intercept) | 3.80 |  |  | 3.30 | 4.30 |
| Kcal labels*Familiarity | -0.05 | 0.05 | .309 | -0.19 | 0.08 |
| Proportional pricing*Familiarity | 0.09 | 0.06 | .094 | -0.05 | 0.23 |
| (Intercept) | 3.71 |  |  | 3.22 | 4.20 |
| Kcal labels*Education (university) | 0.08 | 0.08 | .365 | -0.14 | 0.29 |
| Proportional pricing*Education (university) | 0.01 | 0.08 | .941 | -0.21 | 0.22 |
| (Intercept) | 3.71 |  |  | 3.34 | 4.08 |
| Kcal labels*Ethnicity (non-white) | 0.14 | 0.11 | .216 | -0.08 | 0.37 |
| Proportional pricing*Ethnicity (non-white) | 0.04 | 0.12 | .734 | -0.19 | 0.26 |
| (Intercept) | 3.67 |  |  | 3.18 | 4.16 |
| Kcal labels*Gender (Woman) | -0.03 | 0.08 | .703 | -0.24 | 0.18 |
| Proportional pricing*Gender (Woman) | -0.09 | 0.08 | .287 | -0.30 | 0.12 |
| **Sandwich shop spend** | | | | | |
| (Intercept) | 10.19 |  |  | 8.10 | 12.28 |
| Kcal labels*BMI | 0.00 | 0.03 | .912 | -0.96 | 0.09 |
| **Proportional pricing*BMI** | **0.10** | **0.04** | **.006** | **0.01** | **0.19** |
| (Intercept) | 5.04 |  |  | 2.44 | 7.63 |
| Kcal labels*Age | 0.00 | 0.01 | .858 | -0.03 | 0.04 |
| Proportional pricing*Age | -0.01 | 0.01 | .365 | -0.05 | 0.02 |
| (Intercept) | 7.55 |  |  | 5.13 | 9.97 |
| Kcal labels*Health | 0.33 | 0.31 | .288 | -0.47 | 1.14 |
| Proportional pricing*Health | -0.03 | 0.31 | .927 | -0.84 | 0.78 |
| (Intercept) | 9.51 |  |  | 7.00 | 12.01 |
| Kcal labels*Sensory | 0.17 | 0.36 | .646 | -0.76 | 1.10 |
| Proportional pricing*Sensory | -0.15 | 0.36 | .671 | -1.08 | 0.77 |
| (Intercept) | 6.82 |  |  | 4.49 | 9.16 |
| Kcal labels*Price | 0.57 | 0.33 | .084 | -0.28 | 1.41 |
| Proportional pricing*Price | 0.24 | 0.32 | .448 | -0.59 | 1.07 |
| (Intercept) | 6.61 |  |  | 3.59 | 9.63 |
| Kcal labels*Weight | 0.02 | 0.26 | .920 | -0.64 | 0.69 |
| Proportional pricing*Weight | -0.24 | 0.26 | .349 | -0.90 | 0.42 |
| (Intercept) | 7.92 |  |  | 5.39 | 10.45 |
| Kcal labels*Familiarity | 0.28 | 0.28 | .319 | -0.44 | 0.99 |
| Proportional pricing*Familiarity | 0.06 | 0.28 | .823 | -0.66 | 0.79 |
| (Intercept) | 7.52 |  |  | 4.96 | 10.08 |
| Kcal labels*Education (university) | 0.15 | 0.38 | .691 | -0.84 | 1.14 |
| Proportional pricing*Education (university) | 0.21 | 0.38 | .591 | -0.79 | 1.20 |
| (Intercept) | 7.46 |  |  | 4.86 | 10.06 |
| Kcal labels*Ethnicity (non-white) | 0.22 | 0.61 | .718 | -1.36 | 1.80 |
| Proportional pricing*Ethnicity (non-white) | -0.01 | 0.61 | .982 | -1.59 | 1.57 |
| (Intercept) | 7.31 |  |  | 4.68 | 9.94 |
| Kcal labels*Gender (Woman) | 0.19 | 0.38 | .613 | -0.79 | 1.18 |
| Proportional pricing*Gender (Woman) | -0.78 | 0.38 | .042 | -1.77 | 0.21 |
| **Fast food spend** | | | | | |
| (Intercept) | 7.23 |  |  | 6.50 | 7.95 |
| Kcal labels*BMI | -0.03 | 0.01 | .015 | -0.06 | -0.00 |
| Proportional pricing*BMI | 0.01 | 0.01 | .512 | -0.02 | 0.04 |
| (Intercept) | 5.69 |  |  | 4.87 | 6.51 |
| Kcal labels*Age | -0.00 | 0.00 | .693 | -0.01 | 0.01 |
| Proportional pricing*Age | -0.01 | 0.00 | .024 | -0.02 | 0.00 |
| (Intercept) | 6.44 |  |  | 5.57 | 7.31 |
| Kcal labels*Health | 0.05 | 0.12 | .645 | -0.25 | 0.35 |
| Proportional pricing*Health | -0.15 | 0.12 | .208 | -0.44 | 0.15 |
| (Intercept) | 7.15 |  |  | 6.30 | 8.00 |
| Kcal labels*Sensory | -0.14 | 0.13 | .271 | -0.48 | 0.19 |
| Proportional pricing*Sensory | -0.16 | 0.13 | .226 | -0.49 | 0.18 |
| (Intercept) | 6.21 |  |  | 5.45 | 6.72 |
| Kcal labels*Price | 0.01 | 0.11 | .948 | -0.29 | 0.30 |
| Proportional pricing*Price | -0.03 | 0.11 | .782 | -0.32 | 0.26 |
| (Intercept) | 5.83 |  |  | 4.97 | 6.70 |
| Kcal labels*Weight | 0.00 | 0.09 | .963 | -0.23 | 0.24 |
| Proportional pricing*Weight | -0.15 | 0.09 | .097 | -0.38 | 0.08 |
| (Intercept) | 6.43 |  |  | 5.59 | 7.28 |
| Kcal labels*Familiarity | -0.24 | 0.10 | .015 | -0.49 | 0.01 |
| Proportional pricing*Familiarity | 0.02 | 0.10 | .808 | -0.24 | 0.29 |
| (Intercept) | 6.41 |  |  | 5.57 | 7.25 |
| Kcal labels*Education (university) | -0.05 | 0.14 | .695 | -0.41 | 0.30 |
| Proportional pricing*Education (university) | 0.13 | 0.14 | .320 | -0.21 | 0.48 |
| (Intercept) | 6.40 |  |  | 5.57 | 7.23 |
| Kcal labels*Ethnicity (non-white) | 0.06 | 0.20 | .768 | -0.46 | 0.58 |
| Proportional pricing*Ethnicity (non-white) | 0.23 | 0.20 | .263 | -0.30 | 0.75 |
| (Intercept) | 6.42 |  |  | 5.59 | 7.24 |
| Kcal labels*Gender (Woman) | 0.09 | 0.14 | .520 | -0.26 | 0.44 |
| Proportional pricing*Gender (Woman) | 0.05 | 0.14 | .693 | -0.30 | 0.41 |

All models in secondary analyses included main effects of kcal labelling condition and pricing condition, covariates and the moderator of interest.

## Supplementary material x: Interaction analyses

|  | **Estimate** | **99% CI** | |
| --- | --- | --- | --- |
|  |  | **Lower** | **Upper** |
| **Effect of weight control food choice motives on calories ordered (each unit increase in weight control FCM is associated with the reported change in kcals)** | | | |
| Calorie labelling | 13.20 | -5.65 | 31.98 |
| No calorie labelling | -16.0 | -36.58 | 4.49 |
| No labelling - labelling | -29.20* | -54.40 | -4.02 |
| **Effect of BMI on coffee shop spend (each unit increase in BMI is associated with the reported change to spend)** | | | |
| Proportional pricing | 0.02* | 0.01 | 0.04 |
| Value pricing | 0.00 | -0.01 | 0.01 |
| Value pricing-Proportional pricing | -0.04* | -0.03 | -0.00 |
| **Effect of BMI on sandwich shop spend (each unit increase in BMI is associated with the reported change to spend)** | | | |
| Proportional pricing | 0.15 | 0.08 | 0.23 |
| Value pricing | 0.05 | -0.00 | 0.11 |
| Value pricing-proportional pricing | -0.10 | -0.19 | -0.01 |

*P<.01

We used the R package ‘emmeans’ to investigate the direction of observed interactions for weight control motives and BMI observed in supplementary material xi. For both calorie labelling (b=13.2, 99% CI -5.65 to 31.98) and no calorie labelling conditions(b=-16.0, 95% CI -36.58 to 4.49), there were no significant differences in calories ordered from the coffee shop in relation to weight control food choice motives, however the slope of weight control food choice motives on calories ordered from the coffee shop was significantly smaller in no-labelling conditions (difference = -29.2, p=.003).

For value pricing conditions, there were no significant differences in coffee shop spend in relation to BMI (b=0.00, 99% CI -0.01 to 0.01). For proportional pricing conditions, each unit increase in BMI was associated with an increase of £0.02 from the coffee shop (b=0.02, 95% CI 0.01 to 0.04). The slope of BMI on coffee shop spend was significantly smaller in value conditions (difference = -0.04, p=.003)

For sandwich shop orders, there were no significant differences in spend in relation to BMI (b=0.05, 99% CI -0.00 to 0.11). For proportional pricing conditions, each unit increase in BMI was associated with an increase of £0.15 from the sandwich shop(b=0.15, 95% CI 0.08 to 0.23). The slope of BMI on sandwich shop spend was significantly smaller in value conditions (difference = -0.10, p=.006)

## Supplementary material xi: Exploratory analyses

|  | **Outcome** | **Variable** | **Adjusted Coeff.** | **Std. Error** | **Sig.** | **95% CI** | |
| --- | --- | --- | --- | --- | --- | --- | --- |
|  |  |  |  |  |  | **Lower** | **Upper** |
|  | **Calorie purchase (kcal) from McDonalds mains**  (F(13,1106) = 15.86, p<.001), adjusted R2 of 0.155. | Intercept | 983.69 |  |  | 863.28 | 1104.10 |
|  |  | Pricing (proportional) | -3.19 | 12.96 | .805 | -28.63 | 22.24 |
|  |  | Labelling (kcal labels) | -38.21 | 12.94 | **.003** | -63.60 | -12.81 |
|  | **Money spent (£) from McDonalds mains**  (F(13,1106) = 10.78, p<.001), adjusted R2 of 0.086 | Intercept | 6.14 |  |  | 5.72 | 6.57 |
|  |  | Pricing (proportional) | 0.15 | 0.05 | **<.001** | 0.07 | 0.24 |
|  |  | Labelling (kcal labels) | -0.09 | 0.05 | .054 | -0.18 | 0.00 |
|  | **Calorie purchase (kcal) from McDonalds sides**  (F(13,1106) = 8.32, p<.001), adjusted R2 of 0.091. | Intercept | 20.84 |  |  | -59.23 | 100.92 |
|  |  | Pricing (proportional) | 54.44 | 8.41 | **<.001** | 37.94 | 70.93 |
|  |  | Labelling (kcal labels) | -15.98 | 8.48 | .060 | -32.62 | 0.66 |
|  | **Money spent (£) from McDonalds sides**  (F(13,1106) = 10.77, p<.001), adjusted R2 of 0.110 | Intercept | 0.25 |  |  | -0.18 | 0.67 |
|  |  | Pricing (proportional) | 0.41 | 0.05 | **<.001** | 0.32 | 0.50 |
|  |  | Labelling (kcal labels) | -0.08 | 0.05 | **.**085 | -0.17 | 0.01 |

**References**

1. Haws KL, Liu PJ, Dallas SK, Cawley J, Roberto CA. Any Size for a Dollar: The Effect of Any‐Size‐Same‐Price Versus Standard Pricing on Beverage Size Choices. Journal of Consumer Psychology. 2020;30(2):392-401.

2. Leon AC, Heo M. Sample sizes required to detect interactions between two binary fixed-effects in a mixed-effects linear regression model. Computational Statistics & Data Analysis. 2009;53(3):603-8.
